# A Redox Cu(II)-Graphene Oxide Modified Screen Printed Carbon Electrode as a Cost-Effective and Versatile Sensing Platform for Electrochemical Label-Free Immunosensor and Non-enzymatic Glucose Sensor

**DOI:** 10.3389/fchem.2021.671173

**Published:** 2021-05-20

**Authors:** Sopit Phetsang, Duangruedee Khwannimit, Parawee Rattanakit, Narong Chanlek, Pinit Kidkhunthod, Pitchaya Mungkornasawakul, Jaroon Jakmunee, Kontad Ounnunkad

**Affiliations:** ^1^Department of Chemistry, Faculty of Science, Chiang Mai University, Chiang Mai, Thailand; ^2^National Institute of Technology, Nagaoka College, Niigata, Japan; ^3^Division of Chemistry, School of Science, Walailak University, Nakhon Si Thammarat, Thailand; ^4^Synchrotron Light Research Institute (Public Organization), Nakhon Ratchasima, Thailand; ^5^Environmental Science Research Center, Faculty of Science, Chiang Mai University, Chiang Mai, Thailand; ^6^Center of Excellence for Innovation in Chemistry, Faculty of Science, Chiang Mai University, Chiang Mai, Thailand; ^7^Research Center on Chemistry for Development of Health Promoting Products from Northern Resources, Chiang Mai University, Chiang Mai, Thailand; ^8^Center of Excellence in Materials Science and Technology, Chiang Mai University, Chiang Mai, Thailand

**Keywords:** copper, graphene oxide, immunosensor, electrochemistry, sensor, glucose, immunoglobulin G, screen-printed carbon electrode

## Abstract

A novel copper (II) ions [Cu(II)]-graphene oxide (GO) nanocomplex-modified screen-printed carbon electrode (SPCE) is successfully developed as a versatile electrochemical platform for construction of sensors without an additionally external redox probe. A simple strategy to prepare the redox GO-modified SPCE is described. Such redox GO based on adsorbed Cu(II) is prepared by incubation of GO-modified SPCE in the Cu(II) solution. This work demonstrates the fabrications of two kinds of electrochemical sensors, i.e., a new label-free electrochemical immunosensor and non-enzymatic sensor for detections of immunoglobulin G (IgG) and glucose, respectively. Our immunosensor based on square-wave voltammetry (SWV) of the redox GO-modified electrode shows the linearity in a dynamic range of 1.0–500 pg.mL^−1^ with a limit of detection (LOD) of 0.20 pg.mL^−1^ for the detection of IgG while non-enzymatic sensor reveals two dynamic ranges of 0.10–1.00 mM (sensitivity = 36.31 μA.mM^−1^.cm^−2^) and 1.00–12.50 mM (sensitivity = 3.85 μA.mM^−1^.cm^−2^) with a LOD value of 0.12 mM. The novel redox Cu(II)-GO composite electrode is a promising candidate for clinical research and diagnosis.

## Introduction

Changes in levels of some metabolites, immunogens, or substances in the human body fluids are found and associated with humoral immunological responses against abnormality in health, virus infections, and many diseases (Rama and Costa-García, 2016; Norfun et al., 2017; Putnin et al., 2018). The high levels of some chemicals or metabolites in the human body are specifically due to each disease. Such biomolecular substances can be employed as indicative and clinically relevant biomarkers for medical diagnostics. For instance, a high glucose level in blood is caused by diabetes (Vargas et al., 2019). Elevated levels of immunoglobulins G and M (IgG and IgM) are observed after the infection with new coronavirus 2019 (SARS-CoV-2) for 14 days (Yakoh et al., 2021). In addition, prostate specific antigen (PSA) is detected in men who suffer with prostate cancer (Han et al., 2017; Li and Ma, 2017). These substances can be commonly and frequently used as the target biomarkers for diagnosis of the diseases (Rama and Costa-García, 2016; Han et al., 2017; Li and Ma, 2017; Norfun et al., 2017; Putnin et al., 2018; Vargas et al., 2019; Yakoh et al., 2021). The overexpression of the biomarkers generally occurs at trace levels (Kuntamung et al., 2021). Consequently, high-performance sensing devices are required for the quantitative detections of the biomarkers (Rama and Costa-García, 2016; Norfun et al., 2017; Putnin et al., 2018; Kuntamung et al., 2021). By the detections of the biomarkers, diagnosis before disease severe situation can lead to the successful medical treatments of the health problems, improving the patient survival rates (Condrat et al., 2020). Based on the specific bioreaction and biorecognition (enzyme-substrate and antibody-antigen), many traditional assays have been used for the determination of the biomarkers such as biosensing assays using enzymes (Vargas et al., 2019), enzyme-linked immunosorbent assays (ELISA) (Albright et al., 2016), chemiluminescence immunoassays (Tanaka and Matsunaga, 2000), and surface plasmon resonance (SPR) immunoassays (Dong et al., 2008; Pothipor et al., 2018). Although these methods have high sensitivity and accuracy, they are time-consuming, operated with high cost, and complicated (Jumpathong et al., 2016). Electrochemical sensors have been received increasing attention due to its easy assembly, low-cost, rapid detection, and compatibility for miniaturization (Cruz et al., 2014). The devices present great advantages in determining the target analytes in complicated systems. They also offer low limits of detections (LODs) and high sensitivity (Khristunova et al., 2020).

In recent years, the electrochemical immunosensors have been employed in detecting the many kinds of biomarker proteins for the disease and virus-infection diagnoses (Rama and Costa-García, 2016; Norfun et al., 2017). With some exceptional advantages such as cost-effective instrumentation, less complexity in operation, and fast detection, label-free immunosensors have attracted research development in the diagnoses (Zhang et al., 2008, 2017; Qiu et al., 2010; Jumpathong et al., 2016; Li et al., 2016; Tabrizi et al., 2016; Wang and Ma, 2018; Chanarsa et al., 2020; Kuntamung et al., 2021). The immunosensors are also constructed with no complex fabrication process, leading to convenience of use (Norfun et al., 2016, 2017; Putnin et al., 2018; Wang and Ma, 2018; Zhao et al., 2018; Kuntamung et al., 2021). Moreover, they are actually constructed without non-specific adsorption of the biomarkers in human body fluid, resulting in high selectivity and sensitivity (Putnin et al., 2018; Wang and Ma, 2018). In addition, instead of enzyme based biosensors, many sensors without use of enzymes have been intensively developed for detection of disease-related target analytes (Sridhar and Park, 2018; Li et al., 2019; Wang S. et al., 2020). They are constructed with electrocatalytic materials such as nanometals (Lee et al., 2018; Liu et al., 2019; Chinnadayyala et al., 2021), metal nano-oxides (Zhou et al., 2020; Dong et al., 2021), and nanocomposites (Li et al., 2019; Ramachandran et al., 2019; Wang S. et al., 2020). Such materials offer higher environmental stability (Lee et al., 2018; Ramachandran et al., 2019; Wang S. et al., 2020; Zhou et al., 2020), giving the stable detection performances. Interestingly, these non-enzymatic sensors present acceptable and comparable analytical performances in terms of LOD, sensitivity, and selectivity (Li et al., 2019; Ramachandran et al., 2019; Wang S. et al., 2020). The quantitation of the target substances can be carried out with the recorded amperometric, potentiometric, impedimetric, or conductometric signals (Dawan et al., 2013; Soldatkin et al., 2013; Kim et al., 2017; Zhao et al., 2018).

There are many kinds of electroactive materials employed to construct a sensitive and simple electrochemical sensors such as conducting polymers (Tabrizi et al., 2016; Pothipor et al., 2018; Putnin et al., 2018; Chanarsa et al., 2020), metal nanoparticles (NPs) (Hoa et al., 2015; Jeong et al., 2018; Tran et al., 2018; Dilmac and Guler, 2020), metal oxide nanostructures (Dong et al., 2021; Yang et al., 2021), and carbon nanoarchitectures (Pothipor et al., 2015; Li et al., 2016; Shen and Shen, 2019). Many attempts aim to develop the materials offering the sensors with LODs (Li et al., 2014, 2016; Shen et al., 2015; Zhu et al., 2015; Montes et al., 2016; Tang and Ma, 2016; Zhang et al., 2016; Dong et al., 2017; Han et al., 2017; Barman et al., 2020). Moreover, they are also investigated as multifunctional materials playing many roles in the device performances when they are used to construct electrodes and/or modify electrodes. Two electrochemical devices, namely both supercapacitors and non-enzymatic glucose sensors, are successfully developed using the Cu_x_O/Cu electrodes (Wang S. et al., 2020) and tremella-like NiS/CoS/NiCo_2_S_4_ hierarchical structure (Li et al., 2019). A porous NiO microsphere and Ti_3_C_2_T_x_ hybrid is developed as a bifunctional electrode for uses in supercapacitor and non-enzymatic H_2_O_2_ sensor (Ramachandran et al., 2019). Moreover, layered materials (Wang C. et al., 2020) and conducting polyindole (Marriam et al., 2020) can be used to construct both batteries and supercapacitors. Na-ion batteries and glucose sensor can be elaborated using carbon encapsulated CoS_2_ nanoparticles (NPs) anchored on reduced graphene oxide (rGO) (Sridhar and Park, 2018). Additionally, a report shows a dual-biomarker sensing chip with two working electrodes that are individually modified with different bioactive elements, i.e., enzymes and antibodies for detections of glucose and insulin, respectively (Vargas et al., 2019). There is no report about the materials having many functions that can be used in fabrication of both chemical sensors and immunosensors. Consequently, the investigation of the materials for wide-range applications still remains a crucial challenge. The materials require anti-biofouling and the ability to reduce nonspecific adsorptions of non-target proteins in human serum. To date, many electrochemical platforms are widely used to immobilize antibodies for construction of the immunosensor, including redox hydrogel (Li and Ma, 2017; Tang et al., 2017), CuPdPt nanowire networks (Wen et al., 2018), AuPd NP-multiwalled carbon nanotube (CNT) composite/ferrocenecarboxaldehyde/chitosan hybrid hydrogel (Yin et al., 2018), AgPt nanorings supported on rGO (Wang et al., 2018), and high quality graphene oxide (GO) (Jumpathong et al., 2016; Norfun et al., 2016, 2017). In addition, metals and metal oxides decorated on CNTs, rGO, and GO as efficient electrocatalysts are employed for uses in the non-enzymatic detections (Dhara et al., 2014; Hoa et al., 2015; Ngo et al., 2017; Jeong et al., 2018; Tran et al., 2018; Dilmac and Guler, 2020). Among these nanocarbons, low-cost GO and rGO attracted more interest because of its large electrochemical active surface area, oxygenic functional groups in complexation with metal ions/metal NPs (redox probes) and in the immobilization of the active antibodies, and good electrical conductivity (Wu et al., 2013; Li et al., 2016; Sridhar and Park, 2018; Wang et al., 2018; Barman et al., 2020). Moreover, metal-rGO and metal-GO complexes as redox materials can be eventually used for constructions of immunosensors (Wu et al., 2013; Li et al., 2016; Wang et al., 2018; Barman et al., 2020) and electrocatalysis-based sensors with no enzyme usage (Alizadeh and Mirzagholipur, 2014; Badhulika et al., 2014; Jiang et al., 2014; Hoa et al., 2015; Yazid et al., 2016; Ngo et al., 2017; Sridhar and Park, 2018; Dilmac and Guler, 2020). It is known that although rGO has the higher electric conductivity, GO offers sufficient electrochemical reactivity (Jumpathong et al., 2016; Norfun et al., 2017). Utilization of GO for both sensor types may reduce the reduction step and it would obtain high uptake of metals (redox probes or electrocatalysts), thus giving high device performances. Among many metal nanostructures, copper is particularly interesting material due to its considerable low cost compared to AgNPs and AuNPs. It also reveals high electrical conductivity and good electrocatalytic activity, having great potential for non-enzymatic glucose sensor application (Song et al., 2013; Na et al., 2019). In many cases, the precisely controlled conditions, specific technical skills, complicated instruments, reducing agents, and capping agents are typically required for the synthesis of Cu nanostructures or their nanocomposites to control nanocrystal growth, prevent agglomeration, avoid oxidation, and ensure good dispersibility and stability (Na et al., 2019). Consequently, the Cu(II)/GO nanocomposite is interesting due to its ease of formation and simple preparation, which Cu(II) can offer the catalytic centers for electrooxidation of glucose as well as electrochemical amplification using its redox response in immunosensor.

Herein, a versatile redox Cu(II)/GO platform was simply prepared via an adsorption of Cu(II) ions on a disposable GO-modified screen-printed carbon electrode (SPCE) and successfully employed for the fabrications of label-free electrochemical immunosensor and non-enzymatic glucose sensor, for the first time. The redox Cu(II)/GO-modified SPCE has a dual-function, namely signal amplification in the immunosensor and electrocatalysis in the glucose sensor. Our redox Cu(II)/GO-modified SPCE was further used in the immobilization of the anti-immunoglobulin G (anti-IgG) antibodies for construction the immunosensor. By virtue of the loaded Cu(II) in Cu(II)/GO as the redox probe, the analytical responses of the proposed label-free immunosensor for the sensitive quantification of immunoglobulin G (IgG) were performed by restriction of voltammetric stripping due to the occurrence of immunocomplex formed on the electrode. A Cu(II)/GO-modified SPCE containing Cu(II) species as electrocatalytic centers was used as a sensing electrode for the non-enzymatic quantitative detection of glucose. The Cu(II) can electrocatalyze the oxidation of glucose, producing the current response proportional to glucose concentration. Both sensors preliminarily reveal satisfied sensitivities, selectivities, dynamic ranges, and LODs for detections of IgG and glucose. This bifunctional Cu(II)/GO electrode is a good candidate for many applications in clinical diagnoses such as screening and monitoring diabetes and detecting new coronavirus (COVID-19) infection together with IgM determination (Yakoh et al., 2021).

## Experimental Details

### Chemicals and Materials

Copper (II) nitrate [Cu(NO_3_)_2_, 99.5%] was purchased from Loba Chemie, India. Copper (II) acetate (C_4_H_6_CuO_4_, 98%) was achieved from Carlo Erba reagents (Milan, Italy). Hydrochloric acid (HCl, 37%), sulfuric acid (H_2_SO_4_, 96%), potassium fericyanide{K_3_[Fe(CN)]_6_, 98.5%}, potassium nitrate (KNO_3_, 99%) were purchased from Lab Scan (Poland). Graphite powder (synthetic, size < 20 μm), dopamine hydrochloride (DA, 99.5%), anti-human immunoglobulin G (Fab specific) antibody (anti-IgG, 5.5 mg.mL^−1^) produced in goat, immunoglobulin G (IgG) from human serum (IgG, 4.8 mg.mL^−1^, ≥95%), human serum from human male (AB plasma, USA origin, lot: SLBS6544), myoglobin from human heart (Mb, 2.4 mg.mL^−1^, ≥95%), and phosphate-buffered saline (PBS) tablets (pH 7.4) were ordered from Sigma-Aldrich (Singapore). Nitric acid (HNO_3_, 65%), sodium di-hydrogen phosphate dehydrate (NaH_2_PO_4_·2H_2_O, 98.5%), and L(+)-Ascorbic acid (AA, C_6_H_8_O_6_, 99.7%) were achieved from Merck (Germany). Di-Sodium hydrogen phosphate dehydrate (Na_2_HPO_4_·2H_2_O, 99%) from Scharlau (Spain), glucose (C_6_H_12_O_6_, 99%) from Fluka (Switzerland), hydrogen peroxide (H_2_O_2_, 50%) from AJAX (Australia), potassium permanganate (KMnO_4_, 99%) from Carlo Erba (Italy), and uric acid (C_5_H_4_O_3_N_4_, 98.5%) from Hopkin and Wiliums (USA) were obtained and used as achieved. Bovine serum albumin (BSA, 98%) and interleukin-15 (IL-15, lot: 2381730, ≥98%) were ordered from Merck (Germany) and Millipore (USA), respectively. Sodium acetate (CH_3_COONa·3H_2_O, 99.1%) and acetic acid (CH_3_COOH, 99.7%) were purchased from Fisher Scientific (USA) and RCI Lab Scan (Thailand), respectively. Deionized water (DI water, 18.2 MΩ cm^−1^ at 25) was collected from a purification system (Millipore systems, USA) and used throughout this study. In addition, 100-fold and 50-fold dilution human sera were employed for study of the IgG and glucose determinations in real sample analyses.

### Fabrication of Sensor and Biosensor

**Scheme 1:**
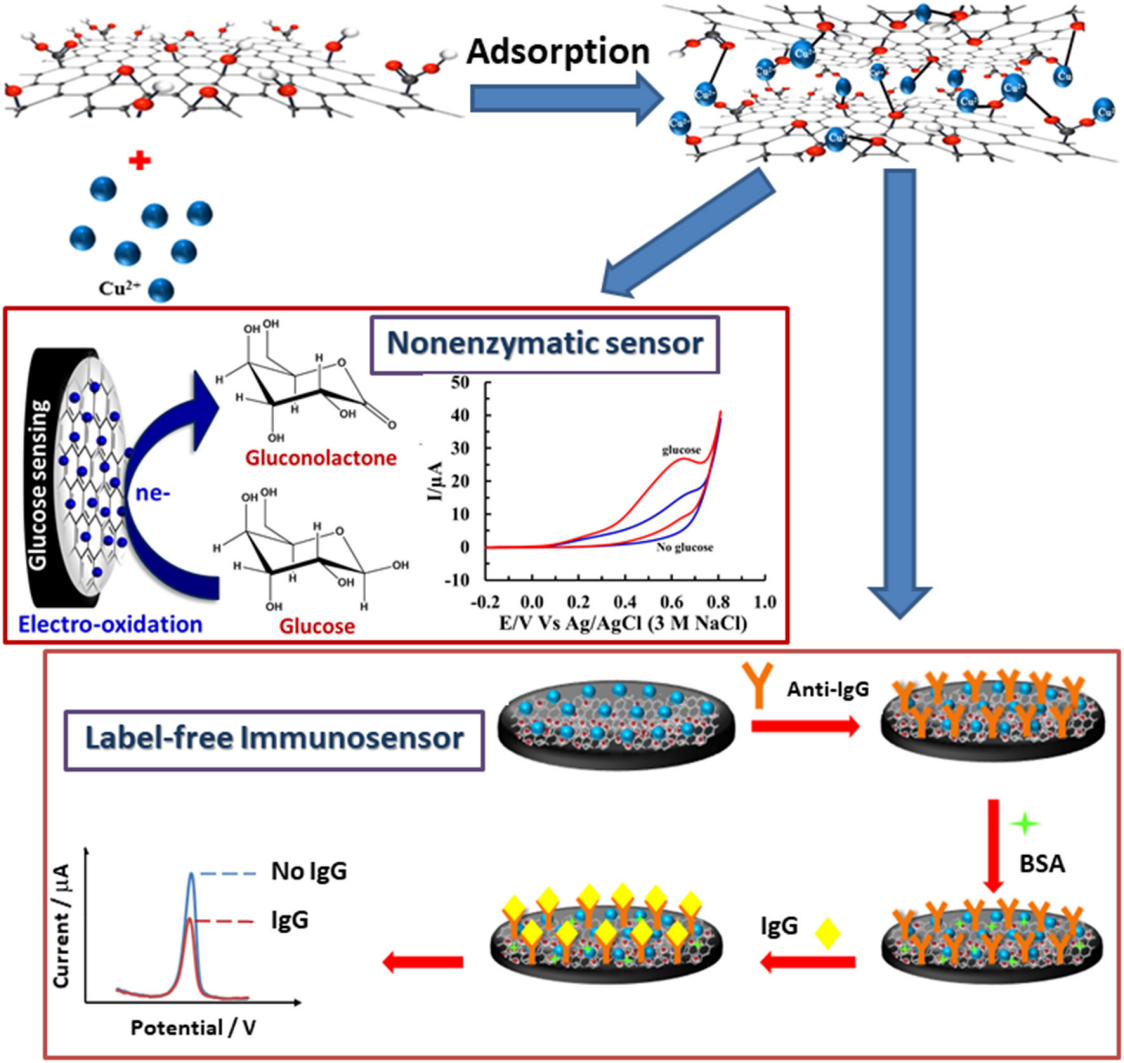
Fabrications of IgG immunosensor and non-enzymatic glucose sensor based on a versatile Cu(II)/GO-modified SPCE.

Graphene oxide (GO) powder achieved from the modified Hummers' process (Pothipor et al., 2015; Norfun et al., 2016) was employed for modification of screen-printed carbon electrodes (SPCEs). Firstly, 5 μL of GO dispersion was added onto the plasma-cleaned SPCE and then the SPCE was dried in the air for 2 h. The GO-modified SPCEs were incubated with Cu(II) ions solution for 70 min and washed with DI water several times. 5.0 mM copper (II) acetate solution and 2.5 mM copper (II) nitrate solution were employed for preparation of Cu(II)/GO-modified SPCEs in detections of IgG and glucose, respectively. Scheme 1 shows the fabrications of both immunosensor and non-enzymatic sensor using the Cu(II)/GO-modified SPCE. For construction of immunosensor, the Cu(II)/GO-modified SPCEs were incubated with 7.0 μL of 50 μg.mL^−1^ anti-IgG antibody solution in a humidity chamber at 4°C for 40 min. After incubation, the modified SPCEs were washed with 0.010 M PBS solution (pH 7.4) several times. To eliminate and block non-specific binding or adsorption, the electrodes were incubated with 7.0 μL of 1.0 wt% BSA in the same condition and washed with the same solution. For non-enzymatic glucose fabrication, the Cu(II)/GO-modified SPCEs were directly employed without any further modification and chemical treatment and the detection of glucose was operated in a basic solution (0.10 M NaOH). Finally, for the immunoassay, anti-IgG/BSA/Cu(II)/GO-modified SPCEs were incubated with 7.0 μL of the IgG solutions at different concentrations in the humidity chamber at 4°C for 40 min and, to remove unbounded IgG molecules, the electrodes were consequently washed with the PBS buffer several times. The responses of Cu(II) ions at the electrodes, after incubation with blank and the IgG solutions, were recorded by square wave voltammetry (SWV) at the potential range from −0.50 to 0.40 V (vs. Ag/AgCl) in 0.20 M acetate buffer (pH 5.5). The electrochemical properties of electrodes and the electrocatalysis of the electrodes toward glucose were studied using cyclic voltammetry (CV). For detection of glucose, the responses of fresh Cu(II)/GO-modified SPCE in basic glucose solutions at different concentrations were undertaken using chronoamperometry at an operating potential of +0.50 V in 0.10 M NaOH solution.

### Apparatus and Instrumentation

The morphologies of the electrode surfaces were studied using a JSM-6335F Field-emission Scanning Electron Microscope (SEM, JEOL, Japan). Energy dispersive X-ray spectroscopy pattern was also recorded by this microscope. In addition, X-ray photoelectron spectroscopy (XPS) technique was employed to analyze elemental composition of prepared electrodes. The experiment was measured at the SUT-NANOTEC-SLRI XAS Beamline (BL5.2) and the SUT-NANOTEC-SLRI XPS Beamline (BL5.3) at the Synchrotron Light Research Institute (Public Organization), Thailand. A electrochemical cell used in this study comprised of a working modified SPCE [Cu(II)/GO- or anti-IgG/BSA/Cu(II)/GO-modified SPCE], a platinum wire (Pt) counter electrode (Niko, Japan), and silver/silver chloride (Ag/AgCl, 3 M NaCl) reference electrode (BASi, USA). The SPCEs were prepared following the procedure from previous reports (Reanpang et al., 2015; Thunkhamrak et al., 2017). Cyclic voltammetry (CV), square-wave voltammetry (SWV), and amperometry were performed on Palmsense 3 and Emstat 3 potentiostats, PalmSens (Netherland).

## Results and Discussion

### Adsorption Study of Cu(II) Ions on GO-Modified SPCE

**Figure 1:**
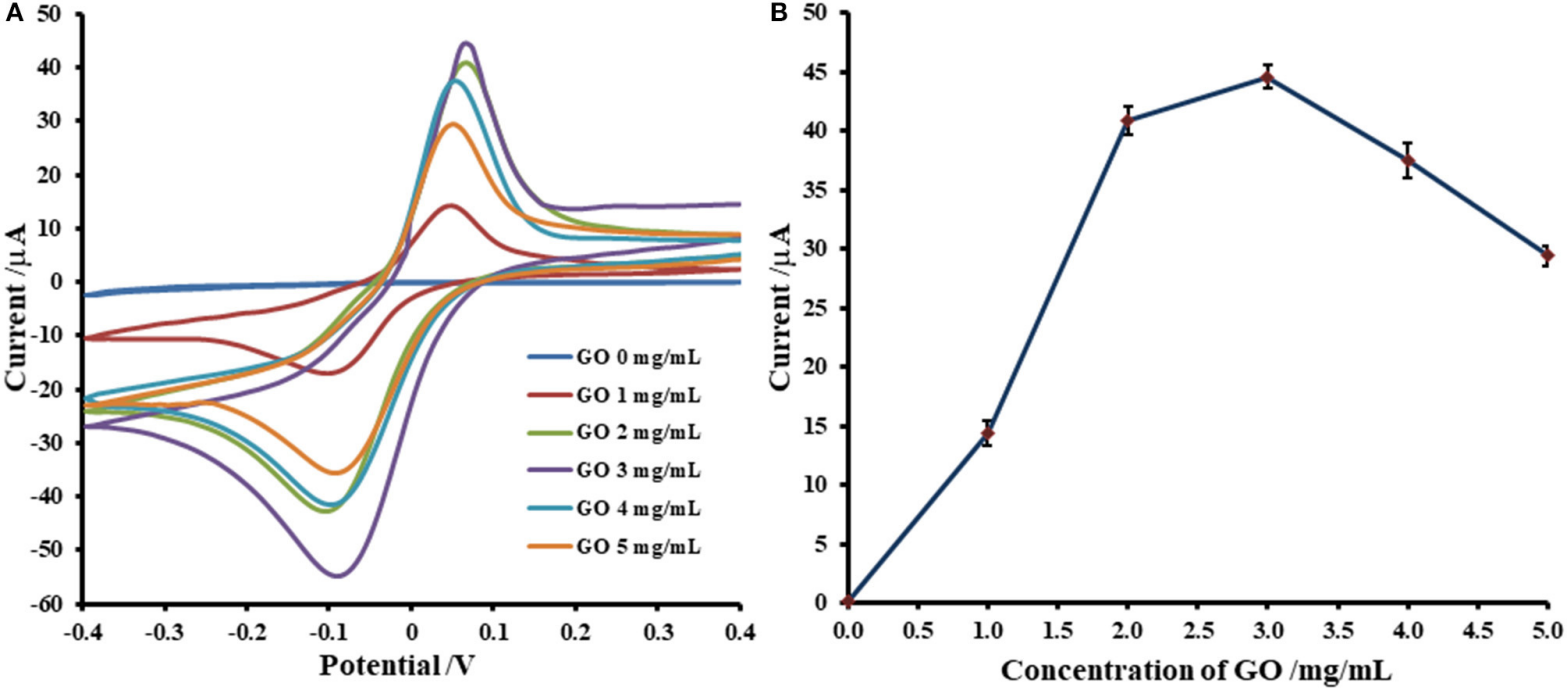
Optimization of GO concentration for Cu(II) ions adsorption on GO-modified SPCE; **(A)** CVs of Cu(II)/GO-modified SPCEs with different GO contents and **(B)** corresponding oxidation peak currents, in 0.20 M acetate buffer (pH 5.5).

**Figure 2:**
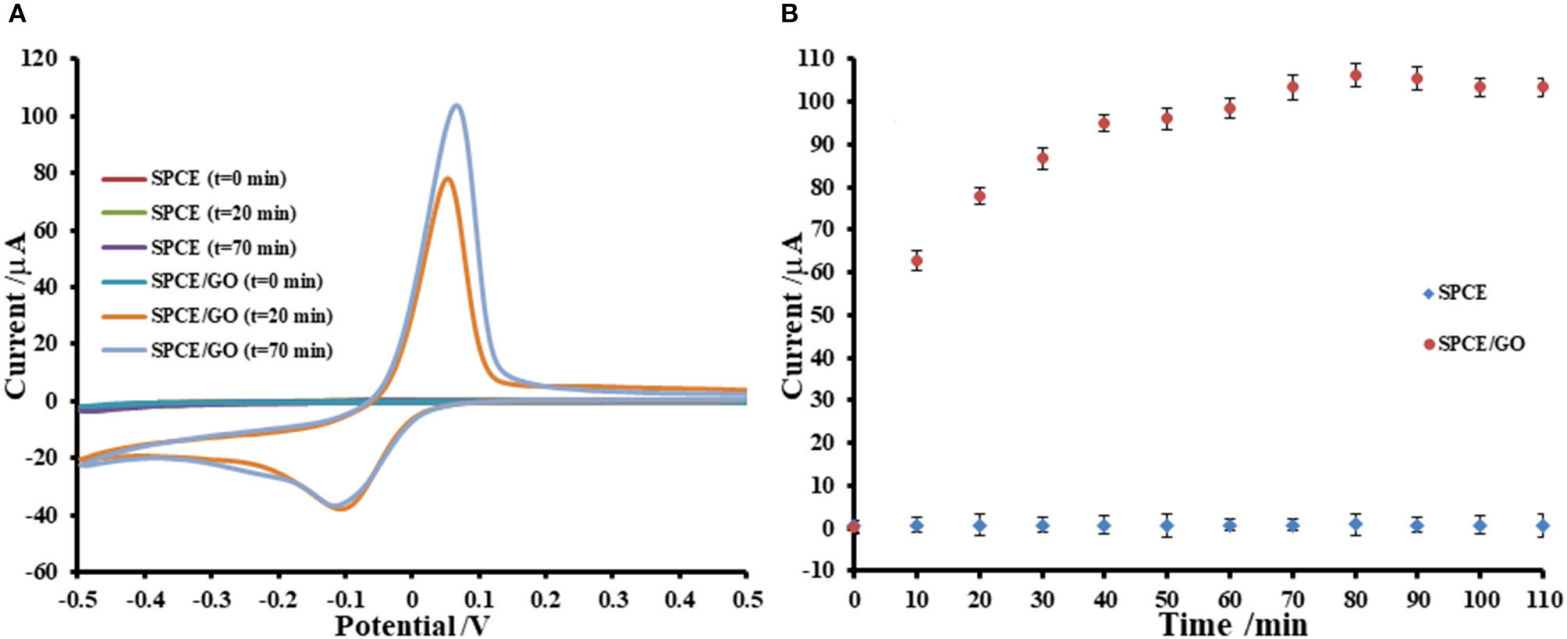
Effect of adsorption period on Cu(II) ions uptake on GO-modified SPCE; **(A)** CVs of Cu(II)/GO-modified SPCEs with different adsorption times and **(B)** corresponding oxidation peak currents, in 0.20 M acetate buffer (pH 5.5).

Adsorption of Cu(II) ions onto GO-modified SPCE is investigated by incubation with 5.0 mM Cu(II) acetate solution. The uptake of Cu(II) in GO-modified SPCE is monitored using CV as shown in Figures 1, 2. In general, the amount of adsorbent (GO) on the electrode is one of the most important parameters, which is needed to be optimized. Although GO is a very good adsorbent for Cu(II) ions (Zhao et al., 2011; Tan et al., 2015; Peng et al., 2017; Ni and Li, 2018), it has low conductivity and low electrochemical reactivity, giving a low redox current response of Cu(II). Thus, high amount of GO sitting on the electrode surface containing high Cu(II) content would not offer the maximum current response of Cu(II). In this study, our GO synthesized by the modified Hummer's method with the triple exfoliation has higher electrochemical reactivity as compared to that of naked SPCE (Jumpathong et al., 2016; Norfun et al., 2017). In order to get the proper Cu(II) uptake as monitored by CV, GO concentration for the modification is investigated in range of 0–5.0 mg.mL^−1^ (0–25 μg). CVs of Cu(II)-adsorbed GO-modified SPCEs with different GO contents (0–25 μg) in contact with 0.20 M acetate buffer (pH 5.5) are recorded as depicted in Figure 1A. The adsorption time is fixed at 5 min. It was found that the current increases to a maximum point and then decreases regarding GO content on the SPCE. As observed in Figure 1B, the GO concentration of 3.0 mg.mL^−1^ (15 μg) offers the maximum current of the oxidation process of Cu(II), suggesting that this GO content would give the best sensitivity of the immunosensing device and the best electrochemical reactivity in the electro-oxidation of glucose, which can be employed in the non-enzymatic detection of glucose. Moreover, the period of time in the adsorption process also affect the amount of Cu(II) in the GO-modified SPCE. Thus, the optimization of the adsorption time is required as shown in Figure 2, of which the CV response of Cu(II) on Cu(II)/GO-modified SPCE is used to track the amount of adsorption. Figure 2A compares the adsorption uptakes in bare SPCE and GO-modified SPCE with different incubation times (0, 20, and 70 min) in Cu(II) solution. It is found that peak responses of copper from all bare SPCEs over long period and GO-modified SPCE with no incubation are not observed, indicating no adsorption of Cu(II) in the naked SPCE. When presence of GO on the platform, the Cu(II) is adsorbed as found that with increasing the period, the response increases. Figure 2B displays plots of the current responses vs. adsorption time for naked SPCE and GO-modified SPCE. There is no significant change in current response over the time from 0 to 110 min for adsorption on bare SPCE whilst for the adsorption on GO-modified SPCE, an increment of the peak response is found until the incubation time of 70 min and then at this point the current intensity starts constant. This suggests that the adsorption of Cu(II) favors and requires GO. The adsorption also needs suitable time to be complete. From this study, the adsorption time of 70 min is chosen for further study as well as construction of the label-free immunosensor and non-enzymatic glucose sensor. This time would offer the best performances of both devices. In addition, the maximum Cu(II) uptake in Cu(II)/GO-modified SPCE (from 3.0 mg.mL^−1^ GO dispersion) at the adsorption time is calculated using the surface coverage equation (Fleming and Bond, 2009). The adsorbed Cu(II) amount is 2.04 ×10^−10^ mole (12.96 ng) and % Cu(II) content in GO (15 μg) on the SPCE is 8.6 ×10^−2^.

### Characterization of Modified SPCEs

**Figure 3:**
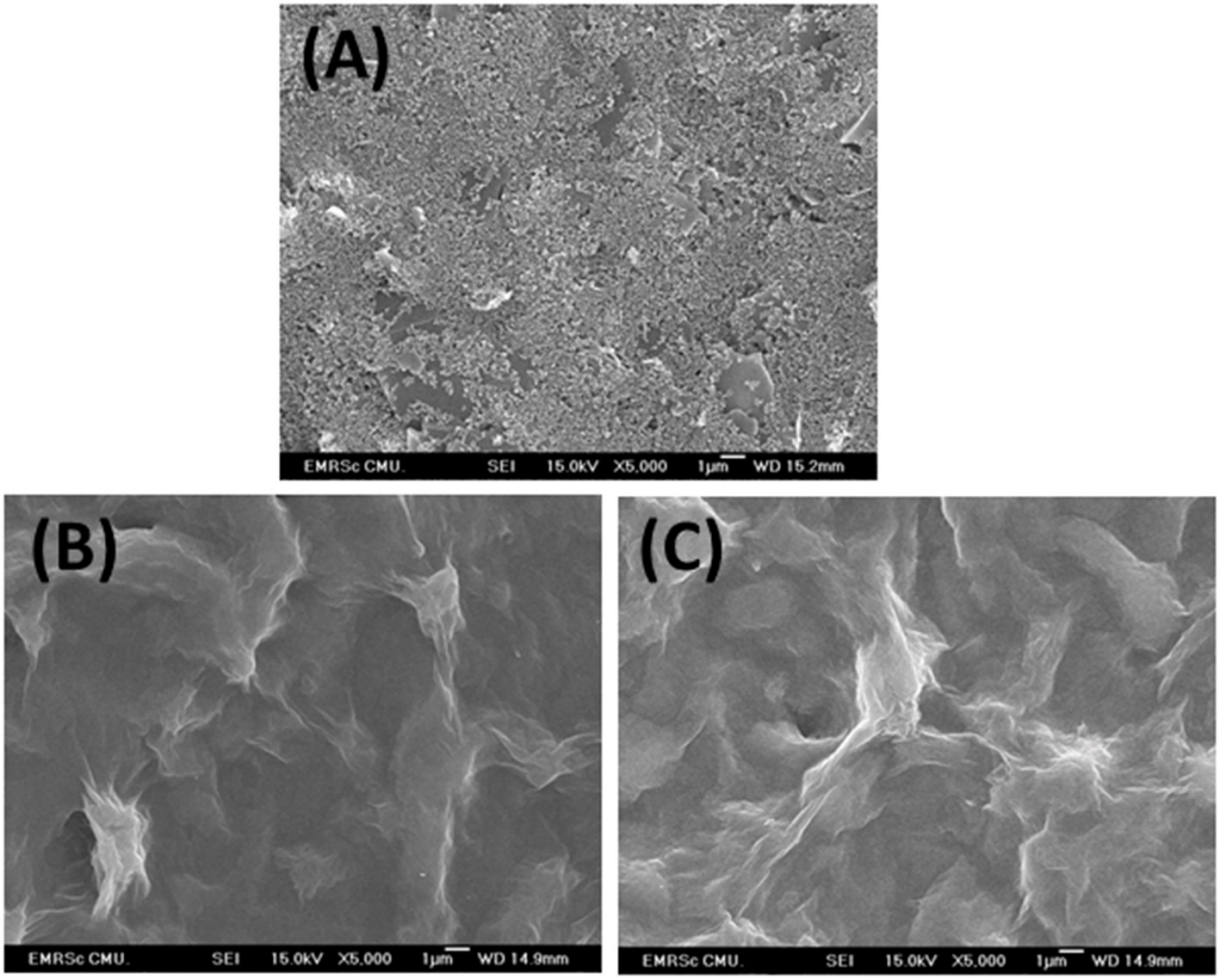
FE-SEM images of bare SPCE **(A)**, GO-modified SPCE **(B)**, and Cu(II)/GO-modified SPCE **(C)**.

As shown in Figure 3, FE-SEM images depict comparison of surfaces of bare SPCE (Figure 3A), GO-modified SPCE (Figure 3B), and Cu(II)/GO-modified SPCE (Figure 3C). Morphology of bare SPCE is rough with some particles of carbons and binder. The mountain-like smooth surface is observed for the two later samples, suggesting full coverage of the electrode with GO. No significant change in the morphology is observed in both samples due to adsorption of Cu(II) ion. To illustrate the presence of copper metal in the GO-modified SPCE, Supplementary Figure 1A shows a FE-SEM image of Cu(II) ion-adsorbed GO-modified SPCE. The wave-like smooth surface of GO characteristic is found, indicating that electrode surface is fully covered with GO. Additionally, Supplementary Figure 1B represents the corresponding EDS spectrum. The spectrum indicates that the surface contains copper metal (3.8 atomic%), carbon (72.3 atomic%), and oxygen (23.9 atomic%). The carbon and oxygen are of GO surface while presence of copper refers to the Cu(II) ions adsorption onto GO-modified SPCE. Supplementary Figure 2 shows XPS spectra of GO- and Cu(II)/GO-modified SPCEs. The simulated spectra show that both surfaces contain some oxygenic functional groups of GO, on which could chelate with the Cu(II) ions, inferring Cu(II)-GO complexes (Muralikrishna et al., 2016). Moreover, the surface would possess charge due to its oxygenic groups in solution. Therefore, the uptake of Cu(II) into GO-modified SPCE by the incubation in Cu(II) solution would involve physical adsorption and the electrostatic interaction as well as the complexation.

**Figure 4:**
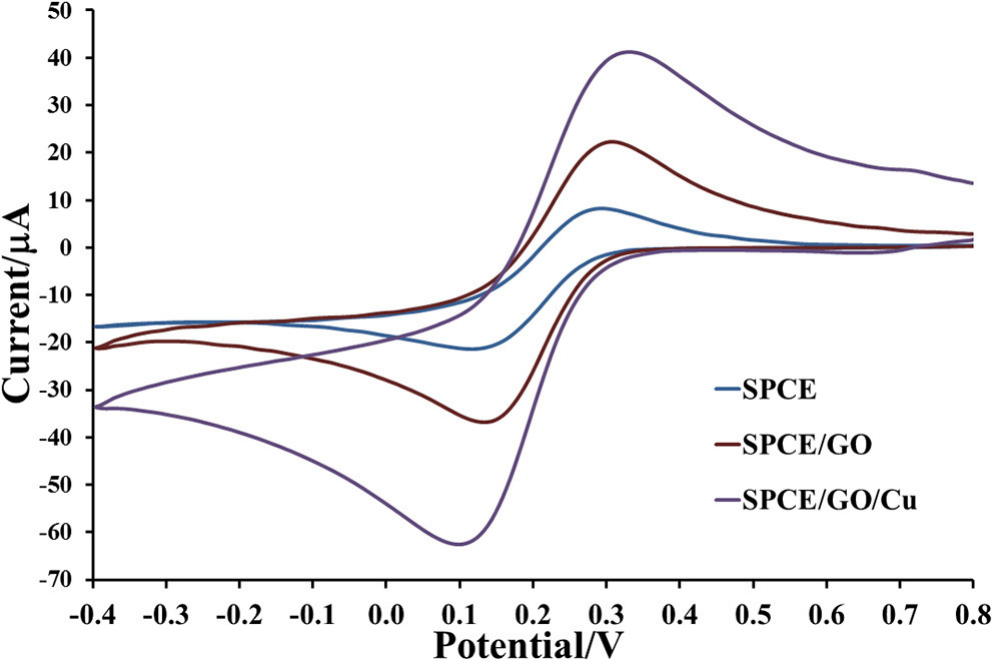
Electrochemical properties of bare SPCE and GO- and Cu(II)/GO-SPCEs in contact with 0.010 M PBS containing 5 mM [Fe(CN)_6_]^3−^.

**Figure 5:**
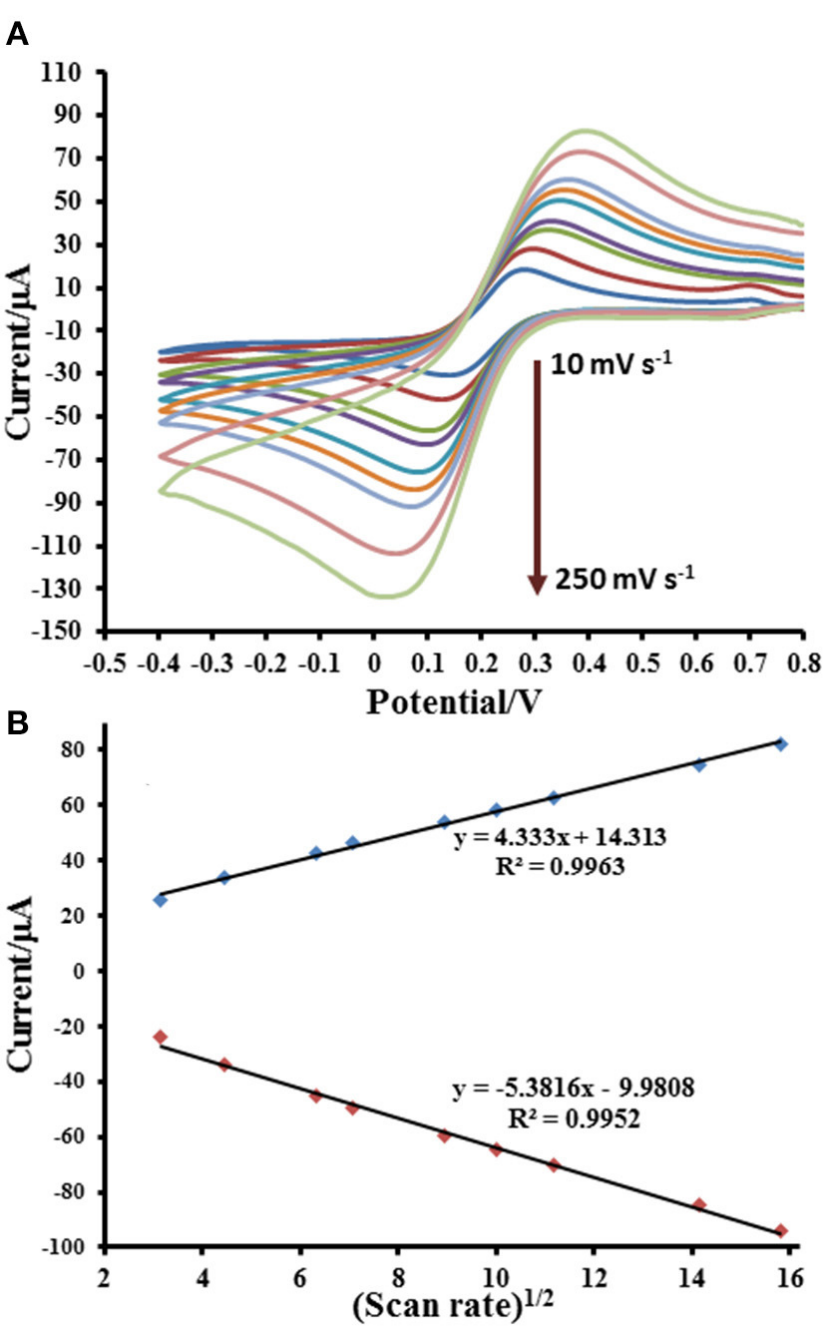
Electrochemical responses for [Fe(CN)_6_]^4−/3−^ process at Cu(II)/GO-modified SPCE; **(A)** CVs and **(B)** related anodic and cathodic peak currents at different scan rates.

The electrochemical properties of prepared electrodes such as current responses (*I*_pa_ and *I*_pc_) and peak-to-peak separation (Δ*E*_P_) were studied by CV in 0.010 M PBS (pH 7.4) solution containing 5.0 mM [Fe(CN)_6_]^3−^ as exhibited in Figure 4. The current response is sequentially improved by modification with GO and Cu(II)/GO complexes, respectively. The oxidation and reduction peak currents of Cu(II)/GO-modified SPCE are improved about 184 and 315%, respectively, from those of naked SPCE. Moreover, the peak separation is also reduced after the modification, indicating that the electron transfer at the electrode is improved and its kinetics is faster. The smallest Δ*E*_P_ value is observed for Cu(II)/GO-modified SPCE, resulting from the fastest electron transfer kinetics (Tsierkezos, 2007). This indicates that Cu(II)/GO offers not only highest electrochemical reactivity but also redox response of redox probe, Cu(II), which can be further used in signal amplification of proposed immunosensor and as catalyst center for non-enzymatic oxidation in the quantitative assay of glucose. Furthermore, CVs of the Cu(II)/GO-modified SPCE in contact with 0.010 M PBS solution containing 5.0 mM [Fe(CN)_6_]^3−^ at different scan rates are recorded as illustrated Figure 5. It is found that the current response increases with increasing the scan rate (Figure 5A) (Tsierkezos, 2007). As seen in Figure 5B, plots of anodic (*I*_pa_) and cathodic (*I*_pc_) peak currents against square root of scan rate show the great linearity, suggesting that the reaction mechanism is diffusion-controlled (Tsierkezos, 2007). It is plausible that the redox reaction at the electrode surface behaves well over the scan rate range. The electrode-to-electrode reproducibility in Cu(II)/GO-modified electrode's production is also assessed by construction of 10 individual electrodes as shown in Supplementary Figure 3. The SWV responses of Cu(II) of the Cu(II)/GO-modified electrodes in contact with 0.2 M acetate buffer (pH 5.5) are examined. It is observed that percent relative standard deviation (% RSD) is of 1.262%, indicating an exceptional electrode fabrication reproducibility.

### Application in Label-Free Immunosensor for IgG Detection

**Figure 6:**
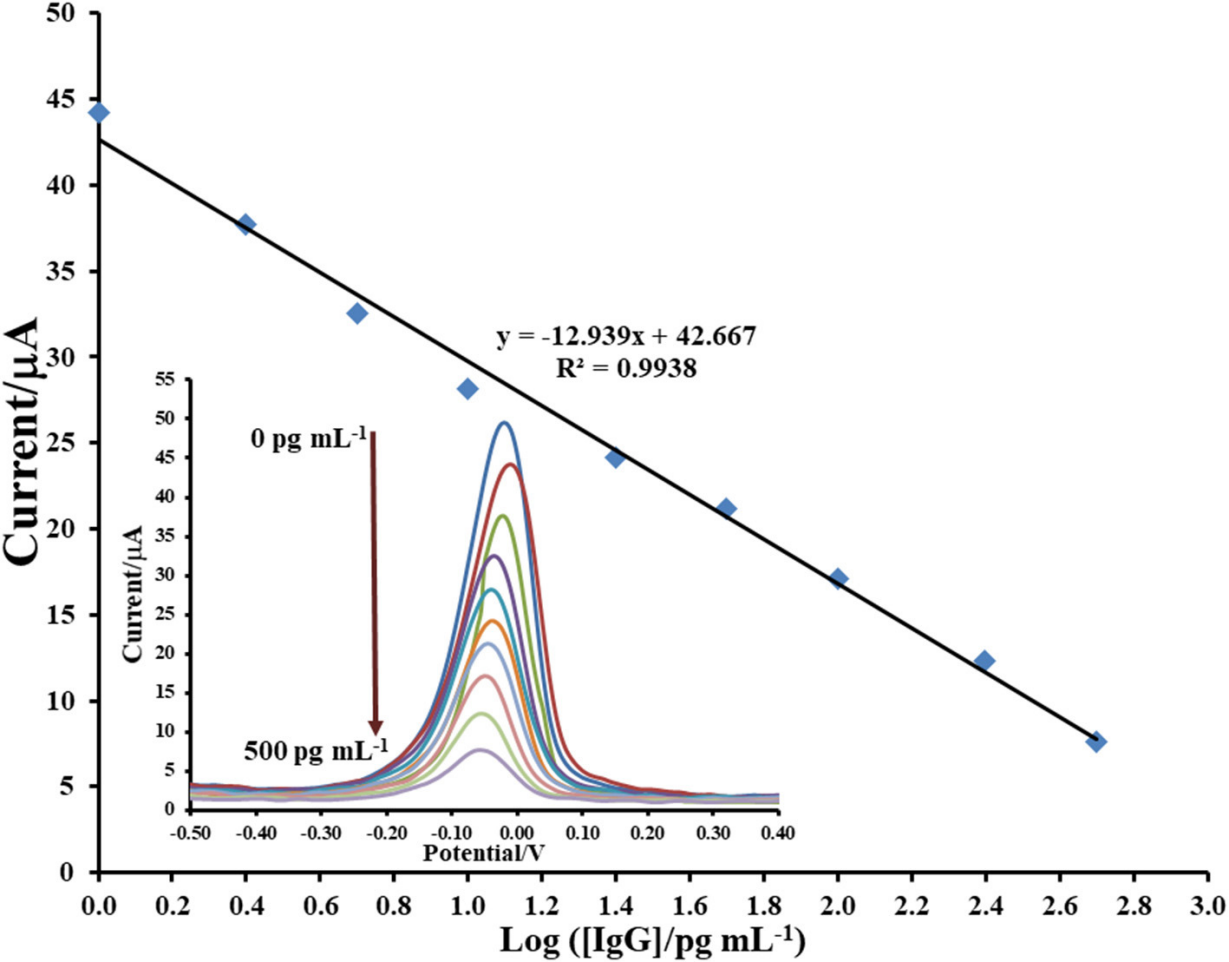
Sensograms and corresponding calibration curve for label-free immunosensing of human IgG using Cu(II)/GO-modified SPCE.

After immunosensor is constructed regarding the Scheme 1 and employing the proposed Cu(II)/GO-modified SPCE, its analytical performances are studied. To construct the calibration curve, the detection of immunoglobulin G (IgG) is tested by incubation of the anti-IgG/BSA/Cu(II)/GO-modified SPCEs' surfaces with the solutions containing different IgG concentrations as illustrated in Figure 6. It is seen that the SWV peaks of the stripping of Cu(II) on the electrodes are located in potential range of *ca*. −0.05–0.00 V. The SWV current response decreases when the IgG concentration is raised. This is due to the restriction of Cu(II) stripping process by the immunoreaction products. The retained response is inversely proportional to the amount of the immunocomplexes. The result reveals the linear logarithmic relationship between current response and IgG concentration from 1 to 500 pg.mL^−1^) with limit of detection (LOD) of 0.20 pg.mL^−1^. The linear equation is *i* (μA) = −12.939log[IgG (pg.mL^−1^)] + 42.667 with a correlation coefficient (*R*^2^) of 0.9938. A comparison of analytical performance of the proposed biosensor with other previous reports is shown in Table 1. LOD and dynamic range of our sensor are comparable and acceptable, which are satisfied in the detection of IgG. Moreover, this immunosensor is much simpler in the detection operation.

**Table 1:** Comparison of our proposed sensors with other reported sensors.

**Immunosensor**	**Detection method**	**Linear range (ng mL^**−1**^)**	**Detection limit (ng mL^**−1**^)**	**References**
OPPy-AuNP/SPE	EIS	0.5–125	2.0 × 10^−2^	Tabrizi et al., 2016
PTH-MB/AuNP/AuE	DPV	10–10^4^	3	Qiu et al., 2010
AuNP/L-cysteine electrode	DPV	0.82–90	25 × 10^−2^	Zhang et al., 2008
Cd^2+^/GP-Fe_3_O_4_/Au@Ag/GCE	Amp	5.0 × 10^−6^−50	2.0 × 10^−6^	Li et al., 2016
GO/SPCE	DPV	2.5–100	1.99	Jumpathong et al., 2016
Cu(II)/GO/SPCE	SWV	1.0 × 10^−3^−0.5	2.0 × 10^−4^	This work
**Glucose sensor**	**Detection method**	**Linear range (mM)**	**Detection limit (μM)**	**References**
**Enzymatic glucose sensor**				
CS/GOx/ZnO/GCE	CV	0.2–5.6	10	Zhou et al., 2013
GOx/MoS_2_/GP/GCE	Amp	2.0–20.0	2.9 × 10^2^	Jeong et al., 2017
GP-CdS-GOx/GCE	CV	2–16	7.0 × 10^2^	Wang et al., 2011
AuNPs/GOx-MWCNTs-PVA/GCE	Amp	0.5–8.0	2.0 × 10^2^	Zhang et al., 2011
PDA/GOx/GP	Amp	0.001–4.7	0.1	Ruan et al., 2013
**Non-enzymatic glucose sensor**				
PDA/CuO-C-dot/SPCE	Amp	0.5–2, 2–5	110, 62.3	Sridara et al., 2020
Cu(II)-C_3_N_4_/MWCNTs/GCE	Amp	0.5 × 10^−3^−12	0.35	Zheng et al., 2018
CuO/TiE	Amp	5.0 × 10^−3^−1.6	2.0	Ji et al., 2014
NiO-TiO_2_/GCE	Amp	2.0 × 10^−3^−2.0	0.7	Rajendran et al., 2018
Pt nanoflowers/MWCNTs/GP/GCE	Amp	1.0–7.0	3.9 × 10^2^	Badhulika et al., 2014
Nafion/CuNPs-N-GP/GCE	Amp	4.0 × 10^−3^−4.5	1.3	Jiang et al., 2014
Cu(II)-GO/SPCE	Amp	0.10–1.0, 1.0–12.5	1.2 × 10^2^	This work

*OPPy, overoxidized polypyrrole; AuNP, gold nanoparticle; SPE, screen printed electrode; EIS, electrochemical impedance spectroscopy; PTH, polythionine; MB, methylene blue; AuE, gold electrode; DPV, differential pulse voltammetry; Cd^2+^, cadmium ion; GP, graphene; Au@Ag, gold and silver core-shell nanoparticles; GCE, glassy carbon electrode; Amp, amperometry; GO, graphene oxide; SPCE, screen printed carbon electrode; Cu(II), copper(II) ion; SWV, square wave voltammetry; CS, chitosan; GOx, glucose oxidase; ZnO, zinc oxide; CV, cyclic voltammetry; MoS_2_, molybdenum disulfide; CdS, cadmium sulfide; MWCNTs, multi-wall carbon nanotubes; PVA, polyvinyl alcohol; PDA, polydopamine; CuO, copper oxide; C-dots, carbon nanodots; C_3_N_4_, graphitic carbon nitride; TiE, titanium substrate electrode; NiO, nickel oxide; TiO_2_, titanium dioxide; Pt, platinum; CuNPs-N-GP, copper nanoparticles decorated nitrogen-doped GP*.

**Figure 7:**
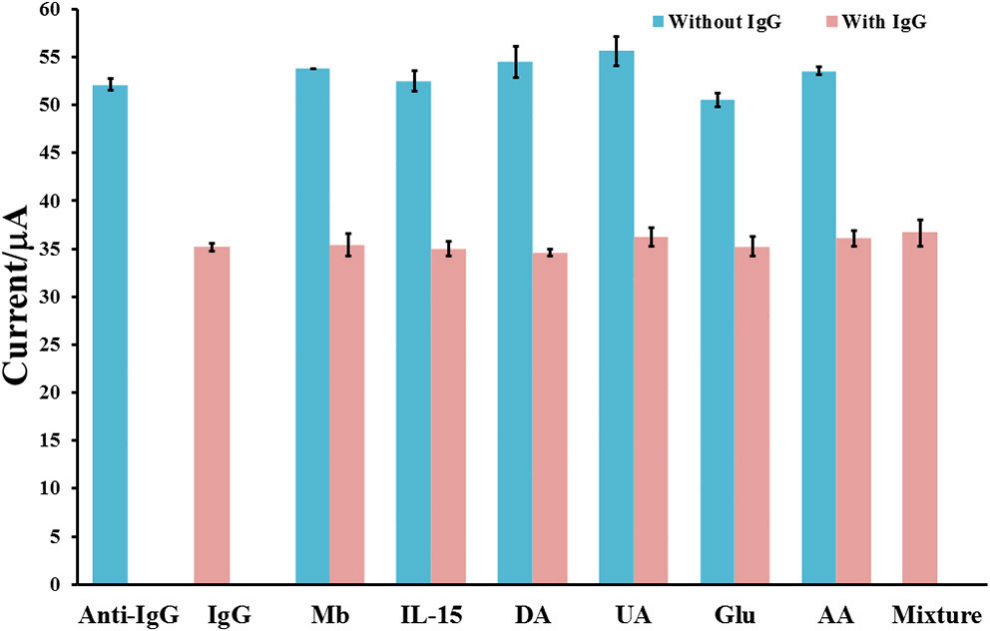
Interference study for label-free immunosensing of human IgG using Cu(II)/GO-modified SPCE.

The inaccuracy in detection of IgG may be caused by interfering substances. Therefore, in this study, myoglobin (MB), interleukin-15 (IL-15), dopamine (DA), uric acid (UA), glucose (Glu), ascorbic acid (AA), and their mixture are used as interferences to examine the specificity and selectivity of the proposed immunosensor as shown in Figure 7. The study is operated with the detections of 2.5 pg mL^−1^ IgG, 250 pg mL^−1^ of each individual interfering substance, mixtures of 2.5 pg mL^−1^ IgG and 250 pg mL^−1^ of each interference, and a mixture of 2.5 pg mL^−1^ IgG and 250 pg mL^−1^ of interference mixture. As compared with the response of blank, the responses of individual interferences determined by our immunosensor are fluctuated and slightly changed (*ca*. 1.9–4.8%). After the sensor surface is incubated with 2.5 pg.mL^−1^ IgG solution, the current response is extremely dropped to lower current response (*ca*. 66.7% of initial value) owing to blockage of redox response by the immunoreaction product. When the detection of IgG is obtained in presence of each interference or the interference mixture, the current responses are significantly different from that of IgG only, indicating high device selectivity. This result shows the good anti-interference ability of the sensor and no non-specific adsorption of interfering substances on the immunosensing surface. Furthermore, to demonstrate the reliability and accuracy, human serum with known amounts of spiked IgG concentrations (10–250 pg mL^−1^) are employed. As summarized in Table 2, the % recoveries and % RSDs are found to be in the ranges of 97.80–102.73% and 0.64–2.03%, respectively. Moreover, the serum used may contain some proteins and salts, which would interfere the detection response. As exhibited in Table 2, they do not affect the biosensor response. The result suggests the satisfactory performance of the proposed immunosensor for real sample analysis. It has the potential to detect IgG with high precision and accuracy.

**Table 2:** Recovery study of IgG in serum sample using the prepared immunosensor.

**Samples**	**Standard of IgG (pg/mL)**	**Found (pg/mL)**	**Recovery (%)**	**RSD (%)**
1	10	10.12	101.2	2.03
2	25	25.68	102.7	0.97
3	50	49.91	99.81	1.56
4	100	97.80	97.80	1.13
5	250	250.6	100.2	0.64

### Application in Non-enzymatic Sensor for Glucose Detection

**Figure 8:**
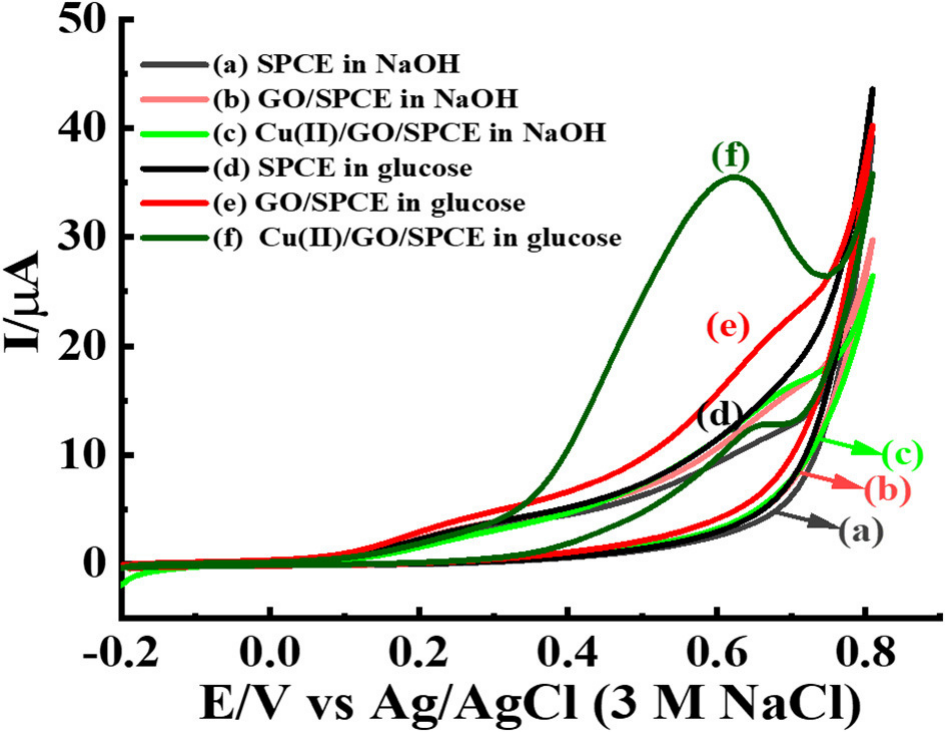
CVs of different modified electrodes; bare SPCE, and GO- and GO/Cu(II)-modified SPCEs in 0.10 M NaOH solution with the absence and presence of 5.0 mM glucose at a scan rate of 50 mV s^−1^.

The Cu(II)/GO-modified SPCE is freshly employed for the electrooxidation of glucose. Figure 8 displays comparison of glucose oxidation at three electrodes, bare SPCE, and GO-, and GO/Cu(II)-modified SPCEs. CVs of the electrodes in contact with 0.10 NaOH solution with absence and presence of 5.0 mM glucose are recorded at the potential range of −0.20 to 0.80 V. The oxidation of glucose can be performed at three electrodes as seen with increased current responses from 0.30 V. Interestingly, Cu(II)/GO-modified SPCE provides the highest oxidation peak of glucose oxidation and the current peak is clearly observed at 0.60 V. Moreover, by the oxidation of 5.0 mM glucose, the current of Cu(II)/GO-modified SPCE is ~3.57-fold and 10-fold higher than those of GO-modified and bare SPCEs, respectively. This indicates good electrocatalysis toward glucose over Cu(II)/GO-modified SPCE. Cu(II) can improve and catalyze the electrochemical oxidation of glucose over GO-modified SPCE as obviously seen with the peak response (+0.60 V) in Figure 8 (curve f). The electrocatalytic activity for glucose oxidation of Cu(II)/GO modified SPCE mainly originates from Cu(II) ion. The catalytic mechanism would be regarding previous proposed reports (Alizadeh and Mirzagholipur, 2014; Jiang et al., 2014; Ji et al., 2014; Sridara et al., 2020; Wang S. et al., 2020). The presence of abundant functional groups and large surface area of GO promotes the favorable adsorption of Cu(II) on the electrode surface which can enhance the electrocatalytic performance. In the alkaline medium, the OH^−^ ion would react with Cu(II) ion to form Cu(OH)_2_ which can catalyze the oxidation reaction of glucose. The current response at 0.60 V is increased with raised glucose concentration (1–10 mM) as shown in Supplementary Figure 4, which can be further used for the development of non-enzymatic glucose sensor. In addition, Supplementary Figure 5 reveals the effect of scan rate on CV response of 5.0 mM glucose at the Cu(II)/GO-modified SPCE. The oxidation current linearly increases with increasing the scan rate, thus indicating that the electrochemical oxidation of glucose at the electrode well behaves in diffusion-controlled process (Tsierkezos, 2007).

**Figure 9:**
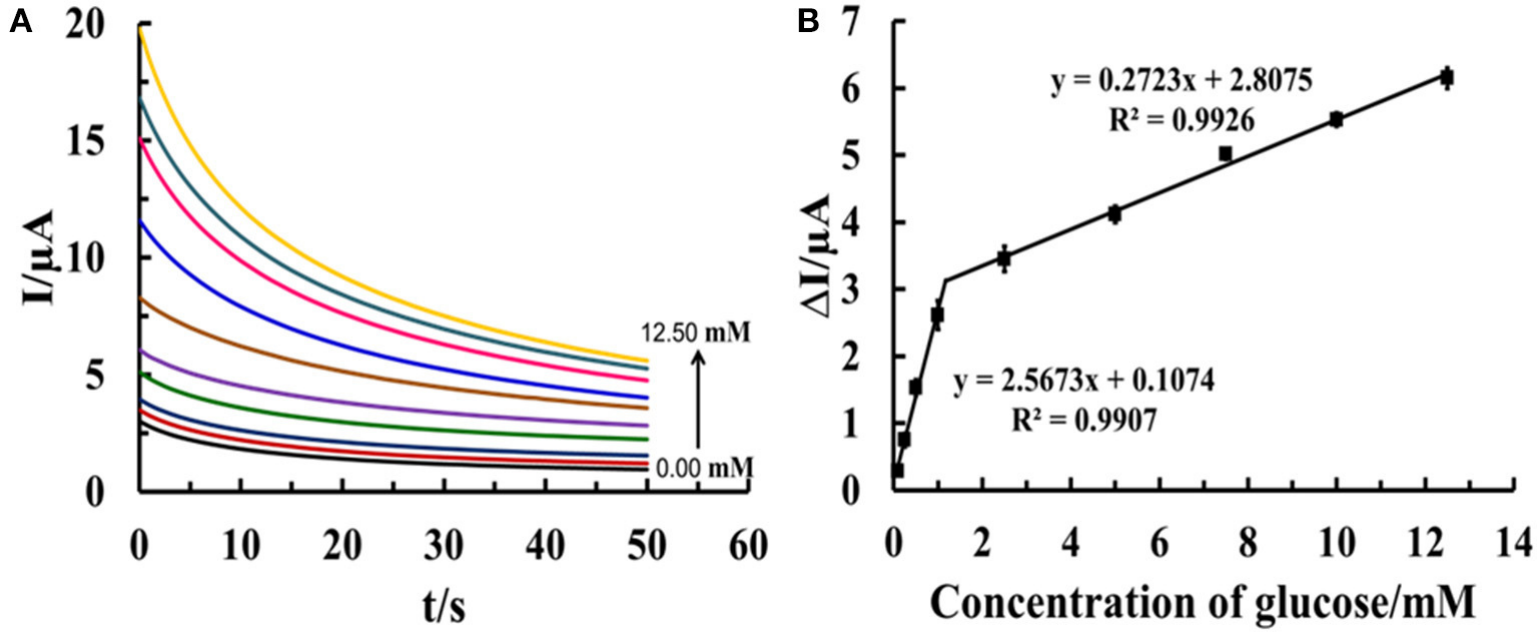
**(A)** Chronoamperograms from different glucose concentrations (0.10–12.50 mM) using Cu(II)/GO-modified electrodes at an operating potential of 0.50 V in 0.10 M NaOH solution and **(B)** the corresponding calibration curves of glucose determination.

For non-enzymatic glucose detection, the sensor based on such electrode is constructed. Chronoamperometric responses of the electro-oxidation of glucose at Cu(II)/GO-modified SPCE-based sensor in contact with different glucose concentrations (0.10–12.50 mM) in 0.10 M NaOH solution are determined at an operating potential of 0.50 V as observed in Figure 9. An increase in current response is significantly observed, after the glucose concentration is raised. The relationship between the steady current and glucose concentration at the Cu(II)/GO modified SPCE (Figure 9B) exhibits two linear ranges of 0.10–1.00 mM (*R*^2^ = 0.9907) and 0.10–12.5 mM (*R*^2^ = 0.9926) with the sensitivities of 36.31 and 3.85 μA mM^−1^ cm^−2^, respectively. The detection limit is estimated to be 0.12 mM (*n* = 3). The result is satisfied in term of the detection of glucose in human blood. Therefore, the sensor can be a good candidate of the analytical tools in use for diabetes diagnosis.

Moreover, to evaluate reproducibility in the glucose oxidation process, the anodic current responses at seven individual Cu(II)/GO-modified electrodes in 0.10 M NaOH containing 5.0 mM glucose are examined as depicted in Supplementary Figure 6. An acceptable % RSD value of 1.60% for the reproducibility is obtained. The 10-day stability of the prepared Cu(II)/GO-modified SPCE is also inspected by measuring the anodic current responses in 0.10 M NaOH containing 5.0 mM glucose. As acquired in Supplementary Figure 7, 95.97% of initial response is found after storage for 10 days. This implies that the Cu(II)/GO-modified electrode for non-enzymatic detection of glucose has high stability. As listed in Table 2, the performance of the developed non-enzymatic sensor vs. available sensors including enzymatic and non-enzymatic sensors are compared. Our non-enzymatic glucose sensor exhibits a comparable LOD value with those of the previous studies involving both kinds of sensors. Furthermore, the non-enzymatic sensor from our study is simpler and cost-effective. Among these biosensors/sensors, it also demonstrates the measurement with no complexity and disposability. The performance of the proposed immunosensor and non-enzymatic sensor, thereby make it very attractive for point-of care (POC) applications.

## Conclusions

A new, simple, and versatile electrochemical platform based on Cu(II)/GO-modified SPCE for both label-free immunosensing detection of IgG and non-enzymatic detection of glucose is successfully developed. The redox Cu(II)/GO-modified electrode is simply prepared with an adsorption of Cu(II) ions onto GO-modified SPCE. Cu(II) on Cu(II)/GO-modified SPCE acts as a redox signal amplifier and a catalytic center toward electrochemical glucose oxidation for immunosensor and non-enzymatic chemical sensor, respectively. Both sensors show prominent analytical performances, which have great potentials to detect IgG and glucose, which would be employed and further developed for clinical diagnoses as well as screening diseases and virus infections, especially COVID-19 infection during the pandemic situation. In addition, the fabrication of the redox Cu(II)/GO-based electrode via the Cu(II) adsorption is facile. According to the benefit of our material, it can be further applied for the immobilization of different antibodies to construct a variety of immunosensors. This platform is a promising candidate in developing the other new immunosensors. With the adsorption strategy for other metal ions and redox molecules, the platform achieved can be further used for fabrication of the multiplex sensors.

## Data Availability Statement

The original contributions presented in the study are included in the article/Supplementary Material, further inquiries can be directed to the corresponding author.

## Author Contributions

DK, SP, NC, and PK: investigation and data curation. PR, PM, and JJ: writing—review and editing. KO: conceptualization, methodology, formal analysis, resources, validation, writing—original draft, writing—review and editing, supervision, project administration, and funding acquisition. All authors contributed to the article and approved the submitted version.

## Conflict of Interest

The authors declare that the research was conducted in the absence of any commercial or financial relationships that could be construed as a potential conflict of interest.

## References

[B1] Albright V. C. (III) Hellmich R. L. Coats J. R. (2016). A review of cry protein detection with enzyme-linked immunosorbent assays. J. Agric. Food Chem. 64, 2175–2189. 10.1021/acs.jafc.5b03766 26949828

[B2] Alizadeh T. Mirzagholipur S. (2014). A nafion-free non-enzymatic amperometric glucose sensor based on copper oxide nanoparticles-graphene nanocomposite. Sens. Actuators B Chem. 198, 438–447. 10.1016/j.snb.2014.03.049

[B3] Badhulika S. Paul R. K. Rajesh Terse T. Mulchandani A. (2014). Nonenzymatic glucose sensor based on platinum nanoflowers decorated multiwalled carbon nanotubes-graphene hybrid electrode. Electroanalysis 26, 103–108. 10.1002/elan.201300286

[B4] Barman S. C. Abu Z. M. Sharifuzzaman M. Ko S. G. Yoon H. Nah J. S. (2020). A polyallylamine anchored amine-rich laser-ablated graphene platform for facile and highly selective electrochemical IgG biomarker detection. Adv. Funct. Mater. 30:1907297. 10.1002/adfm.201907297

[B5] Chanarsa S. Pothipor C. Kungwan N. Ounnunkad K. (2020). A poly(pyrrole-3-carboxylic acid) thin film modified screen printed carbon electrode as highly sensitive and selective label-free electrochemical immunosensing platform. Chiang Mai J. Sci. 47, 530–541.

[B6] Chinnadayyala S. R. Park J. Satti A. T. Kim D. Cho S. (2021). Minimally invasive and continuous glucose monitoring sensor based on non-enzymatic porous platinum black-coated gold microneedles. Electrochim. Acta. 369:137691. 10.1016/j.electacta.2020.137691

[B7] Condrat C. E. Thompson D. C. Barbu M. G. Bugnar O. L. Boboc A. Cretoiu D.. (2020). miRNAs as biomarkers in disease: latest findings regarding their role in diagnosis and prognosis. Cells 9:276. 10.3390/cells9020276 31979244 PMC7072450

[B8] Cruz A. F. D. Norena N. Kaushik A. Bhansali S. (2014). A low-cost miniaturized potentiostat for point-of-care diagnosis. Biosens. Bioelectron. 62, 249–254. 10.1016/j.bios.2014.06.053 25016332

[B9] Dawan S. Wannapob R. Kanatharana P. Limbut W. Numnuam A. Samanman S.. (2013). One-step porous gold fabricated electrode for electrochemical impedance spectroscopy immunosensor detection. Electrochim. Acta. 111, 374–383. 10.1016/j.electacta.2013.08.012

[B10] Dhara K. Stanley J. Ramachandran T. Nair B. G. Babu T. G. S. (2014). Pt-CuO nanoparticles decorated reduced graphene oxide for the fabrication of highly sensitive non-enzymatic disposable glucose sensor. Sens. Actuators B Chem. 195, 197–205. 10.1016/j.snb.2014.01.044

[B11] Dilmac Y. Guler M. (2020). Fabrication of non-enzymatic glucose sensor dependent upon Au nanoparticles deposited on carboxylated graphene oxide. J. Electroanal. Chem. 864:114091. 10.1016/j.jelechem.2020.114091

[B12] Dong Q. Ryu H. Lei Y. (2021). Metal oxide based non-enzymatic electrochemical sensors for glucose detection. Electrochim. Acta. 370:137744. 10.1016/j.electacta.2021.137744

[B13] Dong S. Tong M. Zhang D. Huang T. (2017). The strategy of nitrite and immunoassay human IgG biosensors based on ZnO@ZIF-8 and ionic liquid composite film. Sens. Actuators B Chem. 251, 650–657. 10.1016/j.snb.2017.05.047

[B14] Dong Y. Wilkop T. Xu D. Wang Z. Cheng Q. (2008). Microchannel chips for the multiplexed analysis of human immunoglobulin G–antibody interactions by surface plasmon resonance imaging. Anal. Bioanal. Chem. 390, 1575–1583. 10.1007/s00216-008-1849-7 18251014

[B15] Fleming B. D. Bond A. M. (2009). DC and AC voltammetry of a free-base porphyrin adsorbed onto basal-plane graphite under acidic conditions: an example of a close to ideal reversible two-electron surface-confined redox process at sub-monolayer coverages. Electrochim. Acta 54, 2713–2719. 10.1016/j.electacta.2008.11.042

[B16] Han L. Liu C. M. Dong S. L. Du C. X. Zhang X. Y. Li L. H.. (2017). Enhanced conductivity of rGO/Ag NPs composites for electrochemical immunoassay of prostate-specific antigen. Biosens. Bioelectron. 87, 466–472. 10.1016/j.bios.2016.08.004 27591721

[B17] Hoa L. T. Sun K. G. Hur S. H. (2015). Highly sensitive non-enzymatic glucose sensor based on Pt nanoparticle decorated graphene oxide hydrogel. Sens. Actuators B Chem. 210, 618–623. 10.1016/j.snb.2015.01.020

[B18] Jeong H. Nguyen D. M. Lee M. S. Kim H. G. Ko S. C. Kwac L. K. (2018). N-doped graphene-carbon nanotube hybrid networks attaching with gold nanoparticles for glucose non-enzymatic sensor. Mater. Sci. Eng. C Biomimetic Supramol. Syst. 90, 38–45. 10.1016/j.msec.2018.04.039 29853104

[B19] Jeong J. M. Yang M. Kim D. S. Lee T. J. Choi B. G. Kim D. H. (2017). High performance electrochemical glucose sensor based on three-dimensional MoS_2_/graphene aerogel. J. Colloid Interf. Sci. 506, 379–385. 10.1016/j.jcis.2017.07.061 28750240

[B20] Ji X. Wang A. Zhao Q. (2014). Direct growth of copper oxide films on Ti substrate for nonenzymatic glucose sensors. J. Nanomater. 5:287303. 10.1155/2014/287303

[B21] Jiang D. Liu Q. Wang K. Qian J. Dong X. Yang Z.. (2014). Enhanced non-enzymatic glucose sensing based on copper nanoparticles decorated nitrogen-doped graphene. Biosens. Bioelectron. 54, 273–278. 10.1016/j.bios.2013.11.005 24287416

[B22] Jumpathong W. Jakmunee J. Ounnunkad K. (2016). A sensitive and disposable graphene oxide electrochemical immunosensor for label-free detection of human immunoglobulin G. Anal. Sci. 32, 323–328. 10.2116/analsci.32.323 26960613

[B23] Khristunova E. Dorozhko E. Korotkova E. Kratochvil B. Vyskocil V. Barek J. (2020). Label-free electrochemical biosensors for the determination of Flaviviruses: Dengue, Zika, and Japanese Encephalitis. Sensors 20:4600. 10.3390/s20164600 32824351 PMC7472106

[B24] Kim D.-M. Moon J.-M. Lee W.-C. Yoon J.-H. Choi C. S. Shim Y.-B. (2017). A potentiometric non-enzymatic glucose sensor using a molecularly imprinted layer bonded on a conducting polymer. Biosens. Bioelectron. 91, 276–283. 10.1016/j.bios.2016.12.046 28024285

[B25] Kuntamung K. Sangthong P. Jakmunee J. Ounnunkad K. (2021). Label-free immunosensor for detection of a new lung cancer biomarker, GM2 activator protein, using a phosphomolybdic acid/polyethyleneimine coated gold nanoparticles composite. Analyst 146, 2203–2211. 10.1039/D0AN02149K 33595007

[B26] Lee S. Lee J. Park S. Boo H. Kim H. C. Chung T. D. (2018). Disposable non-enzymatic blood glucose sensing strip based on nanoporous platinum particles. Appl. Mater. Today 10, 24–29. 10.1016/j.apmt.2017.11.009

[B27] Li D. Zhang X. Pei L. Dong C. Shi J. Xu Y. (2019). High-performance supercapacitors and non-enzymatic electrochemical glucose sensor based on tremella-like NiS/CoS/NiCo_2_S_4_ hierarchical structure. Inorg. Chem. Commun. 110:107581. 10.1016/j.inoche.2019.107581

[B28] Li F. Li Y. Dong Y. Jiang L. Wang P. Liu Q.. (2016). An ultrasensitive label-free electrochemical immunosensor based on signal amplification strategy of multifunctional magnetic graphene loaded with cadmium ions. Sci. Rep. 6:21281. 10.1038/srep21281 26880596 PMC4754691

[B29] Li R. Wu K. Liu C. Huang Y. Wang Y. Fang H.. (2014). 4-Amino-1-(3-mercapto-propyl)-pyridine hexafluorophosphate ionic liquid functionalized gold nanoparticles for IgG immunosensing enhancement. Anal. Chem. 86, 5300–5307. 10.1021/ac500024n 24803006

[B30] Li W. Ma Z. (2017). Conductive catalytic redox hydrogel composed of aniline and vinyl-ferrocene for ultrasensitive detection of prostate specific antigen. Sens. Actuators B Chem. 248, 545–550. 10.1016/j.snb.2017.04.021

[B31] Liu S. Liu B. Gong C. Li Z. (2019). A nanoporous Cu-Ag thin film at the Cu-Ag-Zn alloy surface by spontaneous dissolution of Zn and Cu in different degrees as a highly sensitive non-enzymatic glucose sensor. Electrochim. Acta 320:134599. 10.1016/j.electacta.2019.134599

[B32] Marriam I. Wang Y. Tebyetekerwa M. (2020). Polyindole batteries and supercapacitors. Energy Storage Mater. 33, 336–359. 10.1016/j.ensm.2020.08.010

[B33] Montes R. Céspedes F. Baeza M. (2016). Highly sensitive electrochemical immunosensor for IgG detection based on optimized rigid biocomposites. Biosens. Bioelectron. 78, 505–512. 10.1016/j.bios.2015.11.081 26667092

[B34] Muralikrishna S. Cheunkar S. Lertanantawong B. Ramakrishnappa T. Nagaraju D. H. Surareungchai W.. (2016). Graphene oxide-Cu(II) composite electrode for non-enzymatic determination of hydrogen peroxide. J. Electroanal. Chem. 776, 59–65. 10.1016/j.jelechem.2016.06.034

[B35] Na W. Lee J. Jun J. Kim W. Kim Y. Jang J. (2019). Highly sensitive copper nanowire conductive electrode for nonenzymatic glucose detection. J. Ind. Eng. Chem. 69, 358–363. 10.1016/j.jiec.2018.09.050

[B36] Ngo Y.-L. T. Hoa L. T. Chung J. S. Hur S. H. (2017). Multi-dimensional Ag/NiO/reduced graphene oxide nanostructures for a highly sensitive non-enzymatic glucose sensor. J. Alloy Compd. 712, 742–751. 10.1016/j.jallcom.2017.04.131

[B37] Ni L. Li Y. (2018). Role of graphene oxide in mitigated toxicity of heavy metal ions on Daphnia magna. RSC Adv. 8, 41358–41367. 10.1039/C8RA09135H PMC9091651 35559328

[B38] Norfun P. Jumpathong W. Kungwan N. Jakmunee J. Ounnunkad K. (2016). Electroanalytical application of screen-printed carbon electrode modified with conductive graphene oxide polyacrylic acid film for label-free detection of human immunoglobulin G. Chem. Lett. 45, 1444–1446. 10.1246/cl.160715

[B39] Norfun P. Suree N. Kungwan N. Punyodom W. Jakmunee J. Ounnunkad K. (2017). Electrochemical detection of human interleukin-15 using a graphene oxide-modified screen-printed carbon electrode. Anal. Lett. 50, 1112–1125. 10.1080/00032719.2016.1216123

[B40] Peng W. Li H. Liu Y. Song S. (2017). A review on heavy metal ions adsorption from water by graphene oxide and its composites. J. Mol. Liq. 230, 496–504. 10.1016/j.molliq.2017.01.06431838692

[B41] Pothipor C. Kungwan N. Jakmunee J. Ounnunkad K. (2015). A disposable and flexible graphene electrode fabricated by inkjet printing of an aqueous surfactant-free graphene oxide dispersion. Chem. Lett. 44, 800–802. 10.1246/cl.150101

[B42] Pothipor C. Lertvachirapaiboon C. Shinbo K. Kato K. Kaneko F. Ounnunkad K.. (2018). Development of graphene oxide/poly(3,4 ethylenedioxythiophene)/poly(styrene sulfonate) thin film-based electrochemical surface plasmon resonance immunosensor for detection of human immunoglobulin G. Jpn. J. Appl. Phys. 57:02CA07. 10.7567/JJAP.57.02CA07

[B43] Putnin T. Jumpathong W. Laocharoensuk R. Jakmunee J. Ounnunkad K. (2018). A sensitive electrochemical immunosensor based on poly(2-aminobenzylamine) film modified screen-printed carbon electrode for label-free detection of human immunoglobulin G. Artif. Cell Nanomed. Biotechnol. 46, 1042–1051. 10.1080/21691401.2017.1360322 28782437

[B44] Qiu L.-P. Wang C.-C. Hu P. Wu Z.-S. Shen G.-L. Yu R.-Q. (2010). A label-free electrochemical immunoassay for IgG detection based on the electron transfer. Talanta 83, 42–47. 10.1016/j.talanta.2010.08.036 21035641

[B45] Rajendran S. Manoj D. Raju K. Dionysiou D. D. Naushad M. Gracia F.. (2018). Influence of mesoporous defect induced mixed-valent NiO (Ni^2+^/Ni^3+^)-TiO_2_ nanocomposite for non-enzymatic glucose biosensors. Sens. Actuators B Chem. 264, 27–37. 10.1016/j.snb.2018.02.165

[B46] Rama E. C. Costa-García A. (2016). Screen-printed electrochemical immunosensors for the detection of cancer and cardiovascular biomarkers. Electroanalysis 28, 1700–1715. 10.1002/elan.201600126

[B47] Ramachandran R. Zhao C. Rajkumar M. Rajavel K. Zhu P. Xuan W. (2019). Porous nickel oxide microsphere and Ti_3_C_2_T_x_ hybrid derived from metal-organic framework for battery-type supercapacitor electrode and non-enzymatic H_2_O_2_ sensor. Electrochim. Acta 322:134771. 10.1016/j.electacta.2019.134771

[B48] Reanpang P. Themsirimongkon S. Saipanya S. Chailapakul O. Jakmunee J. (2015). Cost-effective flow injection amperometric system with metal nanoparticle loaded carbon nanotube modified screen printed carbon electrode for sensitive determination of hydrogen peroxide. Talanta 144, 868–874. 10.1016/j.talanta.2015.07.041 26452902

[B49] Ruan C. Shi W. Jiang H. Sun Y. Liu X. Zhang X.. (2013). One-pot preparation of glucose biosensor based on polydopamine-graphene composite film modified enzyme electrode. Sens. Actuators B Chem. 177, 826–832. 10.1016/j.snb.2012.12.010

[B50] Shen G. Shen Y. (2019). Covalent Functionalized carbon nanotube with ionic liquid and its application for human immunoglobulin G immunosensor. Int. J. Electrochem. Sci. 14, 7560–7569. 10.20964/2019.08.77

[B51] Shen Y. Zhang Y. Liu M. Liu X. Guo H. Zhang X.. (2015). A simple and sensitive electrochemical immunosensor based on thiol aromatic aldehyde as a substrate for the antibody immobilization. Talanta 141, 288–292. 10.1016/j.talanta.2015.04.004 25966416

[B52] Soldatkin O. O. Peshkova V. M. Saiapina O. Y. Kucherenko I. S. Dudchenko O. Y. Melnyk V. G.. (2013). Development of conductometric biosensor array for simultaneous determination of maltose, lactose, sucrose, and glucose. Talanta 115, 200–207. 10.1016/j.talanta.2013.04.065 24054580

[B53] Song J. Xu L. Zhou C. Xing R. Dai Q. Liu D.. (2013). Synthesis of graphene oxide based CuO nanoparticles composite electrode for highly enhanced nonenzymatic glucose detection. ACS Appl. Mater. Interfaces 5, 12928–12934. 10.1021/am403508f 24182328

[B54] Sridara T. Upan J. Saianand G. Tuantranont A. Karuwan C. Jakmunee J. (2020). Non-enzymatic amperometric glucose sensor based on carbon nanodots and copper oxide nanocomposites electrode. Sensors 20:808. 10.3390/s20030808 32024275 PMC7038693

[B55] Sridhar V. Park H. (2018). Carbon encapsulated cobalt sulfide nano-particles anchored on reduced graphene oxide as high capacity anodes for sodium-ion batteries and glucose sensor. J. Alloy Compd. 764, 490–497. 10.1016/j.jallcom.2018.06.098

[B56] Tabrizi M. A. Shamsipur M. Mostafaie A. (2016). A high sensitive label-free immunosensor for the determination of human serum IgG using overoxidized polypyrrole decorated with gold nanoparticle modified electrode. Mater. Sci. Eng. C Biomimetic Supramol. Syst. 59, 965–969. 10.1016/j.msec.2015.10.093 26652454

[B57] Tan P. Sun J. Hu Y. Fang Z. Bi Q. Chen Y.. (2015). Adsorption of Cu^2+^, Cd^2+^, and Ni^2+^ from aqueous single metal solutions on graphene oxide membranes. J. Hazard. Mater. 297, 251–260. 10.1016/j.jhazmat.2015.04.068 25978188

[B58] Tanaka T. Matsunaga T. (2000). Fully automated chemiluminescence immunoassay of insulin using antibody-protein A-bacterial magnetic particle complexes. Anal. Chem. 72, 3518–3522. 10.1021/ac9912505 10952537

[B59] Tang Z. Fu Y. Ma Z. (2017). Multiple signal amplification strategies for ultrasensitive label-free electrochemical immunoassay for carbohydrate antigen 24-2 based on redox hydrogel. Biosens. Bioelectron. 91, 299–305. 10.1016/j.bios.2016.12.049 28033559

[B60] Tang Z. Ma Z. (2016). Ratiometric ultrasensitive electrochemical immunosensor based on redox substrate and immunoprobe. Sci. Rep. 6:35440. 10.1038/srep35440 27739493 PMC5064308

[B61] Thunkhamrak C. Reanpang P. Ounnunkad K. Jakmunee J. (2017). Sequential injection system with amperometric immunosensor for sensitive determination of human immunoglobulin G. Talanta 171, 53–60. 10.1016/j.talanta.2017.04.058 28551153

[B62] Tran V.-K. Ko E. Geng Y. Kim M. K. Jin G. H. Son S. E.. (2018). Micro-patterning of single-walled carbon nanotubes and its surface modification with gold nanoparticles for electrochemical paper-based non-enzymatic glucose sensor. J. Electroanal. Chem. 826, 29–37. 10.1016/j.jelechem.2018.08.013

[B63] Tsierkezos N. G. (2007). Cyclic Voltammetric studies of ferrocene in nonaqueous solvents in the temperature range from 248.15 to 298.15 K. J. Solut. Chem. 36, 289–302. 10.1007/s10953-006-9119-9

[B64] Vargas E. Teymourian H. Tehrani F. Eksin E. Sánchez-Tirado E. Warren P.. (2019). Enzymatic/immunoassay dual-biomarker sensing chip: towards decentralized insulin/glucose detection. Angew. Chem. Int. Ed. 58, 6376–6379. 10.1002/anie.201902664 30868724

[B65] Wang C. Zhang L. Zhang Z. Zhao R. Zhao D. Ma R.. (2020). Layered materials for supercapacitors and batteries: applications and challenges. Prog. Mater. Sci. 118:100763. 10.1016/j.pmatsci.2020.100763

[B66] Wang H. Ma Z. (2018). Simultaneous detection of multiple tumor markers by label-free electrochemical immunoassay using chip-like glass carbon electrodes. Sens. Actuators B Chem. 256, 402–407. 10.1016/j.snb.2017.10.115

[B67] Wang K. Liu Q. Guan Q. M. Wu J. Li H. N. Yan J. J. (2011). Enhanced direct electrochemistry of glucose oxidase and biosensing for glucose via synergy effect of graphene and CdS nanocrystals. Biosens. Bioelectron. 26, 2252–2257. 10.1016/j.bios.2010.09.043 20947324

[B68] Wang R. Feng J. J. Xue Y. Wu L. Wang A. J. (2018). A label-free electrochemical immunosensor based on AgPt nanorings supported on reduced graphene oxide for ultrasensitive analysis of tumor marker. Sens. Actuators B Chem. 254, 1174–1181. 10.1016/j.snb.2017.08.009

[B69] Wang S. Jiang L. Hu J. Wang O. Zhan S. Lu Y. (2020). Dual-functional Cu_x_O/Cu electrodes for supercapacitors and non-enzymatic glucose sensors fabricated by femtosecond laser enhanced thermal oxidation. J. Alloy. Compd. 815:152105. 10.1016/j.jallcom.2019.152105

[B70] Wen Y. Yuan J. Chen J. Zhao Y. Niu Y. Yu C. (2018). Amperometric myeloperoxidase immunoassay based on the use of CuPdPt nanowire networks. Microchim. Acta 185:55. 10.1007/s00604-017-2563-y 29594375

[B71] Wu Y. Xu W. Wang Y. Yuan Y. Yuan R. (2013). Silver-graphene oxide nanocomposites as redox probes for electrochemical determination of α-1-fetoprotein. Electrochim. Acta 88, 135–140. 10.1016/j.electacta.2012.10.081

[B72] Yakoh A. Pimpitak U. Rengpipat S. Hirankarn N. Chailapakul O. Chaiyo S. (2021). Paper-based electrochemical biosensor for diagnosing COVID-19: Detection of SARS-CoV-2 antibodies and antigen. Biosens. Bioelectron. 176:112912. 10.1016/j.bios.2020.112912 33358057 PMC7746088

[B73] Yang L. Yang J. Dong Q. Zhou F. Wang Q. Wang Z.. (2021). One-step synthesis of CuO nanoparticles based on flame synthesis: as a highly effective non-enzymatic sensor for glucose, hydrogen peroxide, and formaldehyde. J. Electroanal. Chem. 881:114965. 10.1016/j.jelechem.2020.114965

[B74] Yazid S. N. A. M. Isa I. M. Hashim N. (2016). Novel alkaline-reduced cuprous oxide/graphene nanocomposites for non-enzymatic amperometric glucose sensor application. Mater. Sci. Eng. C 68, 465–473. 10.1016/j.msec.2016.06.006 27524043

[B75] Yin S. Zhao L. Ma Z. (2018). Label-free electrochemical immunosensor for ultrasensitive detection of neuron-specific enolase based on enzyme-free catalytic amplification. Anal. Bioanal. Chem. 410, 1279–1286. 10.1007/s00216-017-0767-y 29247379

[B76] Zhang H. Ma L. Li P. Zheng J. (2016). A novel electrochemical immunosensor based on nonenzymatic Ag@Au-Fe_3_O_4_ nanoelectrocatalyst for protein biomarker detection. Biosens. Bioelectron. 85, 343–350. 10.1016/j.bios.2016.04.100 27183286

[B77] Zhang H. Meng Z. Wang Q. Zheng J. (2011). A novel glucose biosensor based on direct electrochemistry of glucose oxidase incorporated in biomediated gold nanoparticles-carbon nanotubes composite film. Sens. Actuators B Chem. 158, 23–27. 10.1016/j.snb.2011.04.057

[B78] Zhang L. Liu Y. Chen T. (2008). A mediatorless and label-free amperometric immunosensor for detection of h-IgG. Int. J. Biol. Macromol. 43, 165–169. 10.1016/j.ijbiomac.2008.04.010 18533249

[B79] Zhang X. Shen Y. Zhang Y. Shen G. Xiang H. Long X.. (2017). A label-free electrochemical immunosensor based on a new polymer containing aldehyde and ferrocene groups. Talanta 164, 483–489. 10.1016/j.talanta.2016.12.016 28107962

[B80] Zhao G. Li J. Ren X. Chen C. Wang X. (2011). Few-layered graphene oxide nanosheets as superior sorbents for heavy metal ion pollution management. Environ. Sci. Technol. 45, 10454–10462. 10.1021/es203439v 22070750

[B81] Zhao L. Han H. Ma Z. (2018). Improved screen-printed carbon electrode for multiplexed label-free amperometric immuniosensor: addressing its conductivity and reproducibility challenges. Biosens. Bioelectron. 101, 304–310. 10.1016/j.bios.2017.10.041 29107882

[B82] Zheng W. Li Y. Liu M. Tsang C.-S. Lee L. Y. S. Wong K.-Y. (2018). Cu^2+^-doped carbon nitride/MWCNT as an electrochemical glucose sensor. Electroanalysis 30, 1446–1454. 10.1002/elan.201800076

[B83] Zhou F. Wang Q. Huang K. Jiang X. Zou Z. Xiong X. (2020). Flame synthesis of NiO nanoparticles on carbon cloth: An efficient non-enzymatic sensor for glucose and formaldehyde. Microchem. J. 159:105505. 10.1016/j.microc.2020.105505

[B84] Zhou Y. Wang L. Ye Z. Zhao M. Cai H. Huang J. (2013). Mango core inner shell membrane template-directed synthesis of porous ZnO films and their application for enzymatic glucose biosensor. Appl. Surf. Sci. 285, 344–349. 10.1016/j.apsusc.2013.08.058

[B85] Zhu C. Yang G. Li H. Du D. Lin Y. (2015). Electrochemical sensors and biosensors based on nanomaterials and nanostructures. Anal. Chem. 87, 230–249. 10.1021/ac5039863 25354297 PMC4287168

