# Supplementary material for: A Redox Cu(II)-Graphene Oxide Modified Screen Printed Carbon Electrode as a Cost-Effective and Versatile Sensing Platform for Electrochemical Label-Free Immunosensor and Non-enzymatic Glucose Sensor
Source: Front Chem. 2021 May 20;9:671173. doi: 10.3389/fchem.2021.671173 (PMC8172615; doi:10.3389/fchem.2021.671173)
Supplement: Supplementary file 1 [file Data_Sheet_1.docx]

Supplementary Material

**A Redox Cu(II)-Graphene Oxide Modified Screen Printed Carbon Electrode as a Cost-effective and Versatile Sensing Platform for Electrochemical Label-free Immunosensor and Non-enzymatic Glucose Sensor**

**Sopit Phetsang^1,2^, Duangruedee Khwannimit^1,3^, Narong Chanlek^4^, Pinit Kidkhunthod^4^, Parawee Rattanakit^2^, Pitchaya Mungkornasawakul^1,5^, Jaroon Jakmunee^1,6,7^, Kontad Ounnunkad^1,6,7,8*^**

^1^Department of Chemistry, Faculty of Science, Chiang Mai University, Chiang Mai 50200, Thailand

^2^National Institute of Technology, Nagaoka College, 888 Nishikatakai-machi, Nagaoka-shi, Niigata, 940-8532, Japan

^3^Division of Chemistry, School of Science, Walailak University, Nakhon Si Thammarat, 80160, Thailand

^4^Synchrotron Light Research Institute (Public Organization), Nakhon Ratchasima 30000, Thailand

^5^Environmental Science Research Center (ESRC), Faculty of Science, Chiang Mai University, Chiang Mai 50200, Thailand

^6^Center of Excellence for Innovation in Chemistry, Faculty of Science, Chiang Mai University, Chiang Mai 50200, Thailand

^7^Research Center on Chemistry for Development of Health Promoting Products from Northern Resources, Chiang Mai University, Chiang Mai 50200, Thailand

^8^Center of Excellence in Materials Science and Technology, Chiang Mai University, Chiang Mai 50200, Thailand

*** Correspondence:**Corresponding Author
kontad.ounnunkad@cmu.ac.th

suriyacmu@yahoo.com


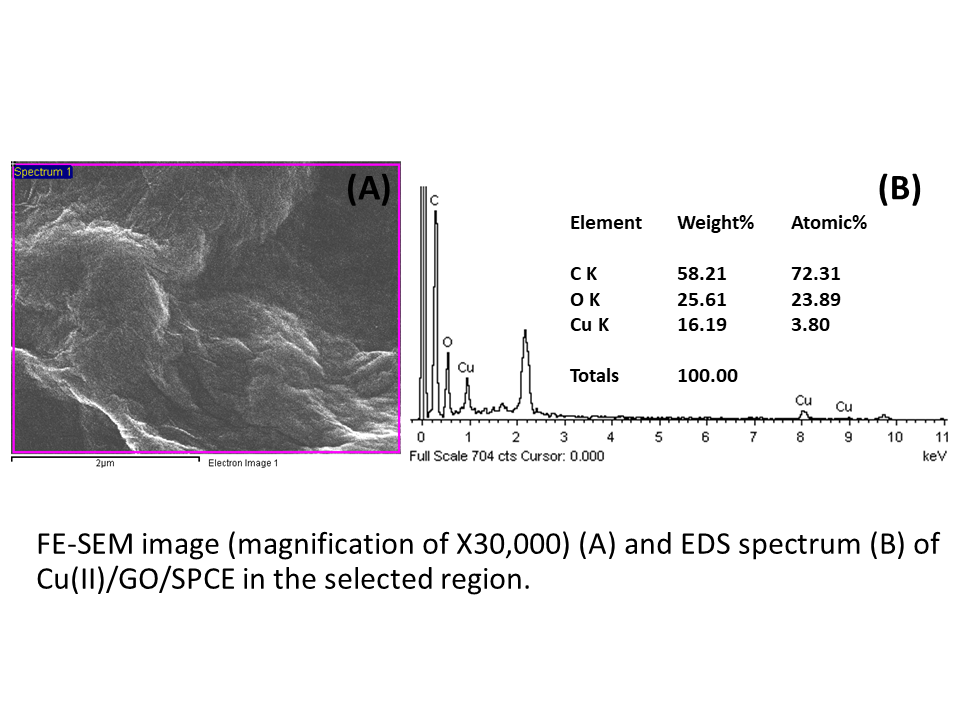


Figure S1. (A) FE-SEM image (magnification of Χ30,000) and (B) EDS spectrum Cu(II)/GO/SPCE.

**
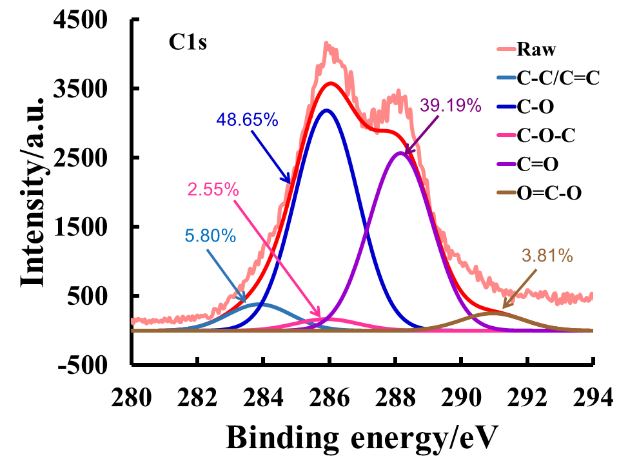

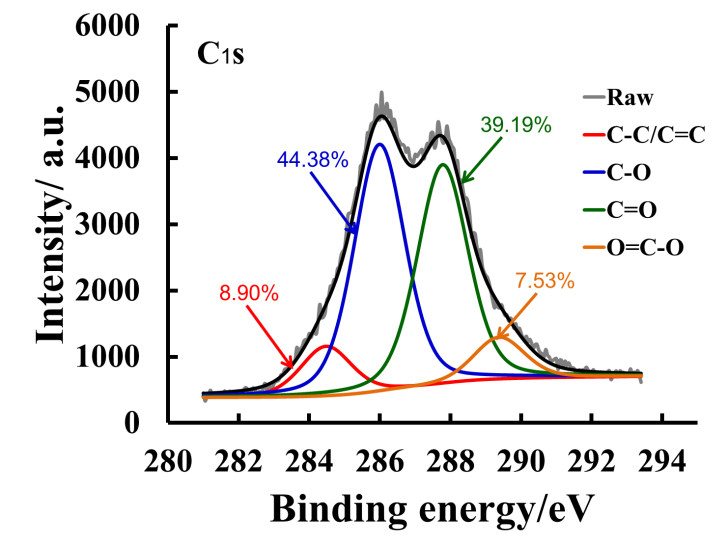
**

**(B)**

**(A)**

**Figure S2.** C1s XPS spectra of (A) GO- and (B) Cu(II)/GO-modified SPCEs.

**
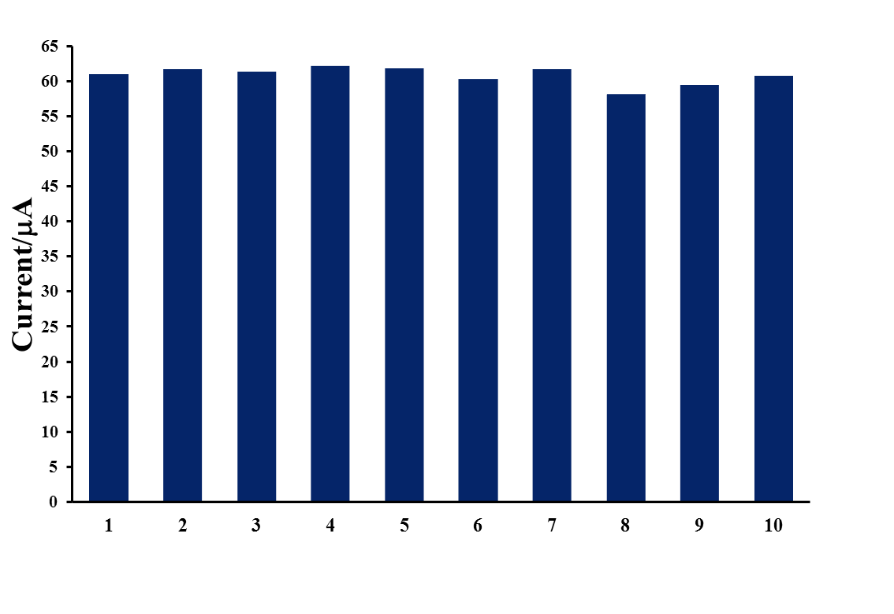
**

**Figure S3** Reproducibility of Cu(II)/GO-modified SPCE; the SWV responses of Cu(II) of ten individual Cu(II)/GO-modified electrodes in 0.2 M acetate buffer (pH 5.5).


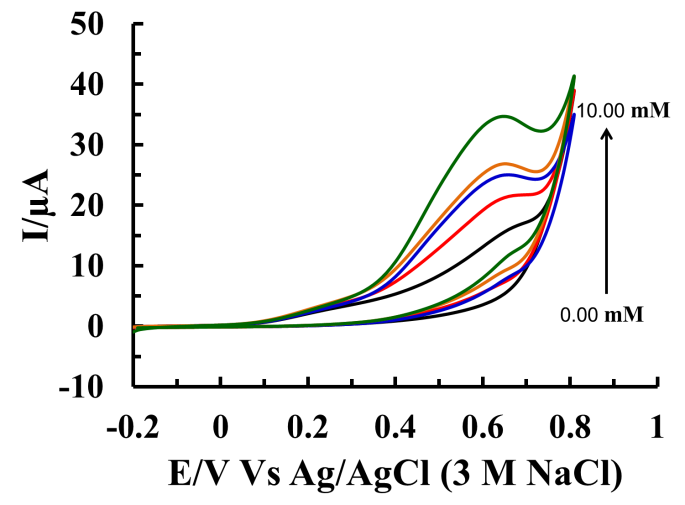


**Figure S4.** CVs of Cu(II)/GO-modified SPCE in 0.10 M NaOH containing different concentrations of glucose (0.0 – 10.0 mM) at a scan rate of 50 mV/s.

**
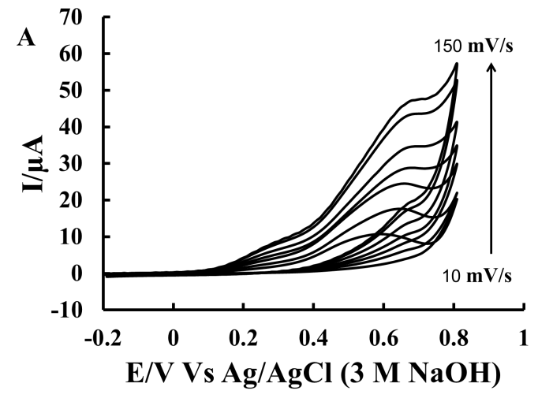

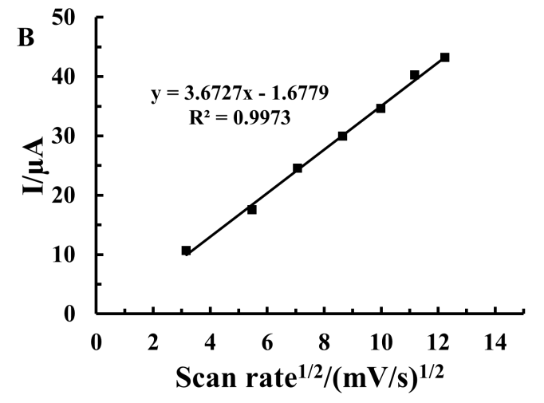
**

**Figure S5.** (A) CVs of Cu(II)/GO-modified SPCE in contact with 0.10 M NaOH solution containing 5.0 mM glucose at different scan rates (10 – 150 mV/s) and (B) the plot of anodic current against square root of scan rate.

**
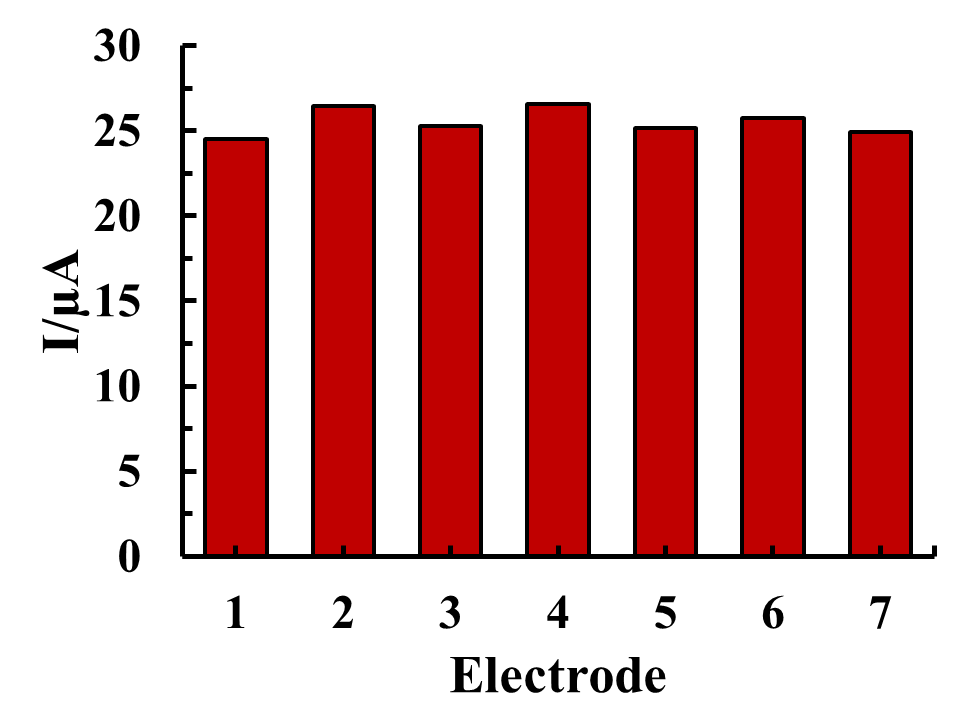
**

**Figure S6.** Reproducibility of Cu(II)/GO-modified electrode; the anodic current responses of glucose at seven individual Cu(II)/GO-modified electrodes in 0.10 M NaOH containing 5.0 mM glucose.


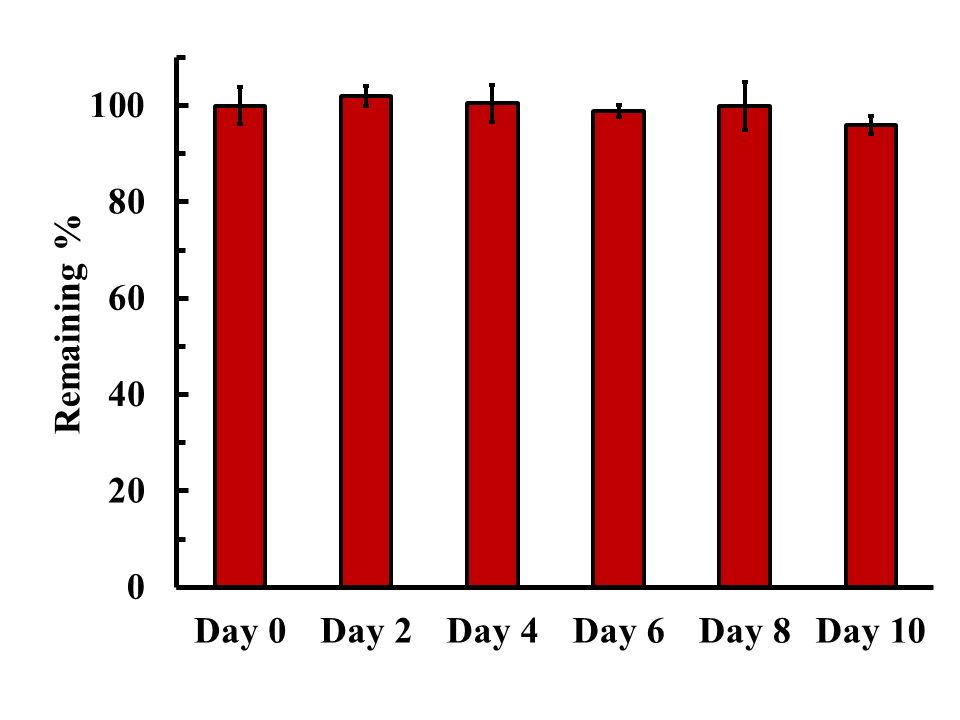


**Figure S7** Stability of Cu(II)/GO-modified electrode over 10 days; the anodic current responses of glucose at Cu(II)/GO-modified electrode in 0.10 M NaOH containing 5.0 mM glucose.
